# Comparative Analysis of mRNA Isoform Expression in Cardiac Hypertrophy and Development Reveals Multiple Post-Transcriptional Regulatory Modules

**DOI:** 10.1371/journal.pone.0022391

**Published:** 2011-07-22

**Authors:** Ji Yeon Park, Wencheng Li, Dinghai Zheng, Peiyong Zhai, Yun Zhao, Takahisa Matsuda, Stephen F. Vatner, Junichi Sadoshima, Bin Tian

**Affiliations:** 1 Department of Cell Biology and Molecular Medicine, University of Medicine and Dentistry of New Jersey, Newark, New Jersey, United States of America; 2 Department of Biochemistry and Molecular Biology, University of Medicine and Dentistry of New Jersey, Newark, New Jersey, United States of America; 3 New Jersey Medical School and Graduate School of Biomedical Science, University of Medicine and Dentistry of New Jersey, Newark, New Jersey, United States of America; Northwestern University, United States of America

## Abstract

Cardiac hypertrophy is enlargement of the heart in response to physiological or pathological stimuli, chiefly involving growth of myocytes in size rather than in number. Previous studies have shown that the expression pattern of a group of genes in hypertrophied heart induced by pressure overload resembles that at the embryonic stage of heart development, a phenomenon known as activation of the “fetal gene program”. Here, using a genome-wide approach we systematically defined genes and pathways regulated in short- and long-term cardiac hypertrophy conditions using mice with transverse aortic constriction (TAC), and compared them with those regulated at different stages of embryonic and postnatal development. In addition, exon-level analysis revealed widespread mRNA isoform changes during cardiac hypertrophy resulting from alternative usage of terminal or internal exons, some of which are also developmentally regulated and may be attributable to decreased expression of Fox-1 protein in cardiac hypertrophy. Genes with functions in certain pathways, such as cell adhesion and cell morphology, are more likely to be regulated by alternative splicing. Moreover, we found 3′UTRs of mRNAs were generally shortened through alternative cleavage and polyadenylation in hypertrophy, and microRNA target genes were generally de-repressed, suggesting coordinated mechanisms to increase mRNA stability and protein production during hypertrophy. Taken together, our results comprehensively delineated gene and mRNA isoform regulation events in cardiac hypertrophy and revealed their relations to those in development, and suggested that modulation of mRNA isoform expression plays an importance role in heart remodeling under pressure overload.

## Introduction

The growth of the heart in mammals takes place at embryonic and postnatal developmental stages, but it can also be induced by physiological or pathological stimuli in the adult [1]. While cardiac growth at early developmental stages mostly involves proliferation of cardiac myocytes, expansion of cell size is chiefly responsible for growth of the adult heart. The latter is also known as cardiac hypertrophy, which is a common component of many cardiac diseases. Cardiac hypertrophy is generally considered as an adaptive mechanism in response to increased mechanical load; but hypertrophied heart under sustained pressure can lead to heart failure [2], [3].

Studies have shown that the expression levels of many genes are regulated during cardiac hypertrophy [4], [5], [6]. A group of genes with low expression in the adult but high expression in the embryo is reactivated in cardiac hypertrophy, a phenomenon widely known as activation of the ‘fetal gene program’. These genes typically play roles in metabolic and contractile functions of the heart [7], [8], and are regulated by a set of transcription factors (TFs) which play roles also in embryonic development, such as NFAT, NFκB, MEF2, GATA4, and SRF [9], [10]. In addition, recent studies have implicated regulation of several microRNAs (miRNAs) in hypertrophy, including miR-1, miR-133, and miR-208 [11], [12], [13]. miRNAs are small non-coding RNAs (∼22 nucleotides) that cause mRNA degradation and/or inhibition of translation by binding to their target sites in mRNAs, mostly in the 3′ untranslated region (3′UTR) [14], [15]. miRNAs have been increasingly found to play important roles in cardiac development and diseases [16], [17], [18], [19].

Mammalian genes frequently express mRNA isoforms resulting from alternative initiation of transcription, alternative splicing (AS), and alternative cleavage and polyadenylation (APA), which are frequently regulated in temporal- and tissue-specific manners [20], [21]. Studies have shown that the AS pattern of the heart is distinct from other tissues [22], [23], [24], and dynamic regulation of AS takes place at the embryonic and postnatal developmental stages of the heart [25], during differentiation of cardiac precursors [26], and in patients with heart failure [27]. Ablation or overexpression of some splicing regulators, such as SC35, ASF/SF2, SRp38, and CELF, have been shown to cause malfunctions in the heart [28], [29], [30], [31].

Recent studies have indicated that APA plays an important role in gene regulation. Over half of the human genes have APA isoforms [32], most of which lead to different 3′UTR lengths. The APA pattern of genes is tissue-specific [33] and is globally regulated in cell proliferation, differentiation, and development [34], [35], [36]. In general, genes tend to express short 3′UTR isoforms more frequently in proliferative or transformed cells than in quiescent or differentiated cells, presumably due to the difference in 3′ end processing activity [37]. Since 3′UTRs typically contain cis elements that inhibit gene expression at the post-transcriptional level, such as AU-rich elements (AREs), GU-rich elements (GREs), and miRNA target sites, regulation of 3′UTR length by APA can impact mRNA metabolism. For example, shortened 3′UTRs in cancer cells have been shown to make mRNAs of a set of oncogenes more stable, leading to higher protein expression [36].

Here we took a genome-wide approach to systematically define genes and pathways regulated in cardiac hypertrophy induced by transverse aortic constriction (TAC), and compared them with those regulated at different stages of embryonic and postnatal development. By exon-level analysis we found that cardiac hypertrophy involves widespread mRNA isoform changes, a fraction of which is developmentally related. Gene Ontology analysis indicated that regulated AS events are biased to genes with functions in cell adhesion and cell morphology, suggesting an important role of AS in remodeling the heart. Our analysis also indicated that downregulated expression of Fox-1 protein during cardiac hypertrophy may be responsible for a set of regulated AS events. Moreover, we found 3′UTRs of mRNAs are generally shortened through APA in hypertrophy, and microRNA target genes are generally de-repressed, suggesting coordinated mechanisms to increase mRNA stability and protein production during hypertrophy. Taken together, our results comprehensively delineated gene regulatory modules at transcriptional and post-transcriptional levels in cardiac hypertrophy and revealed their relations to those executed in development, and suggested that mRNA isoform regulation plays critical roles in remodeling the heart under pressure overload.

## Results

### Comparison of gene expression profiles between cardiac hypertrophy and development defined the fetal gene program

**Figure 1:**
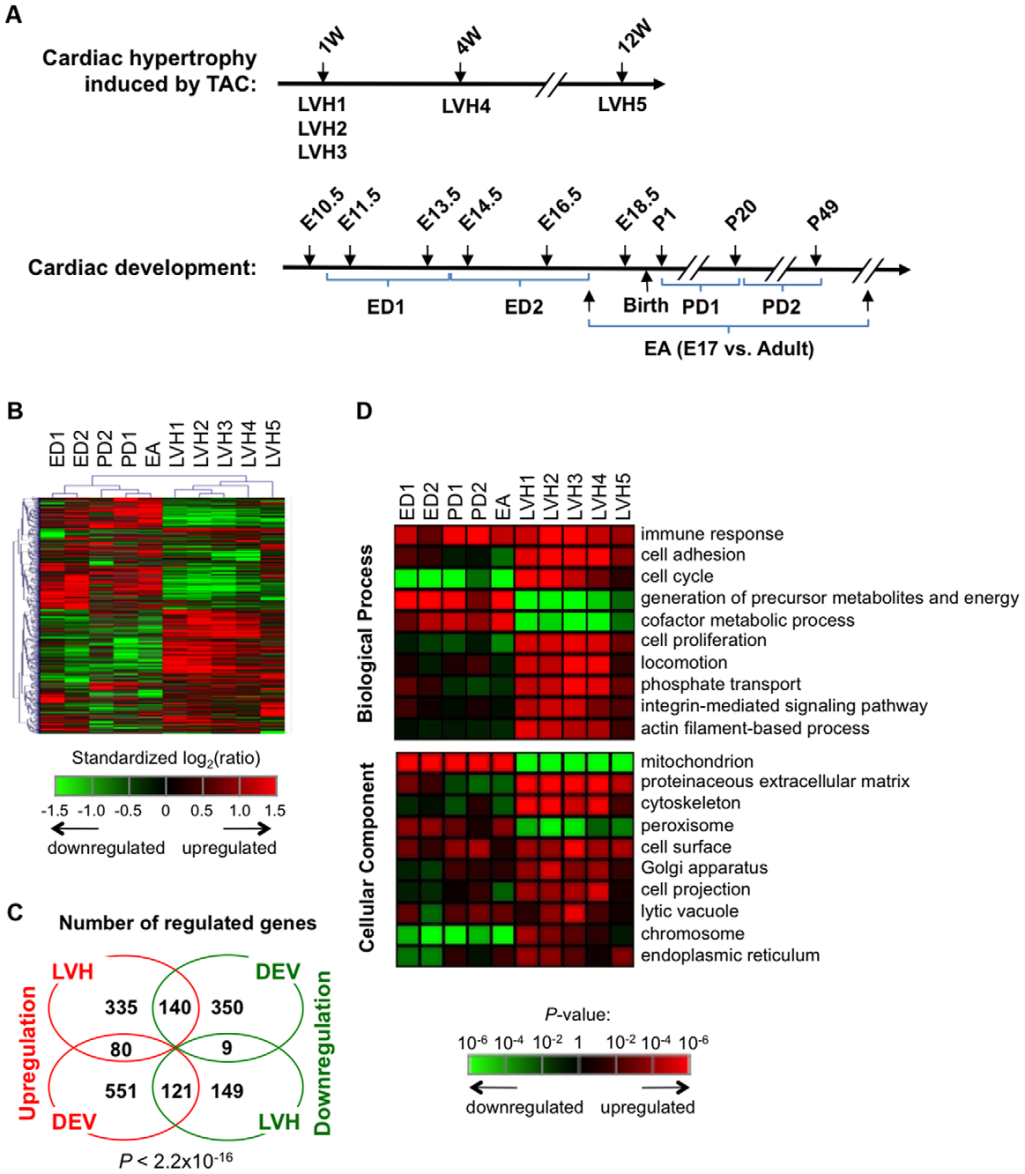
Gene expression profiling in cardiac hypertrophy and development. (**A**) Schematic of datasets used in our analysis. Top, hypertrophy data with TAC period indicated (W = week). All hypertrophy samples were derived from the left ventricle and thus were named LVH. They were compared with Sham samples. Bottom, development samples. E, embryonic day (for mouse samples); P, postnatal day (for rat samples). ED1 is comparison of E10.5 + E11.5 with E13.5 + E14.5 and ED2 is comparison of E13.5 + E14.5 with E16.5 + E18.5. PD1 is comparison of P1 with P20 and PD2 is comparison of P20 with P49. EA is comparison of E17 with adult. E10.5, E11.5, and EA were based on the whole heart, and others were based on the left ventricle. All ratios were derived from expression level at a later development time point divided by that at an earlier point. (**B**) Clustering of genes and samples using gene expression changes. Expression changes are standardized log_2_(ratio) or z, which are shown in a heatmap using the color scale shown at the bottom. A total of 1,945 genes with z >2 in at least one of the samples are shown. Hierarchical clustering is based on Pearson correlation and the average linkage method. (**C**) Venn diagram of genes regulated in development (DEV) and hypertrophy (LVH). Genes were selected from five development datasets, i.e. ED1, ED2, PD1, PD2, and EA, and three 1 W TAC sets, i.e. LVH1, LVH2, and LVH3. A gene was selected if its z is greater than 2 in at least one sample and the direction of its regulation is consistent across samples. Fisher's exact test was used to assess the significance of bias in genes overlapped between DEV and LVH. (**D**) Significant Biological Process (BP, top) and Cellular Component (CC, bottom) terms regulated in hypertrophy. Only top 10 terms are shown for BP and CC. The average p-value of three 1 W TAC samples was used to sort entries. P-values in other samples are also shown for comparison. To remove redundancy, we eliminated GO terms that overlapped by more than 50% of associated genes with another term that had a more significant p-value. P-values are presented in a heatmap according to the color scheme shown in the graph.

To systematically examine gene regulation in short- and long-term cardiac hypertrophy, we used genome-wide exon microarrays to analyze mRNAs expressed in mouse hypertrophied left ventricle (LVH) induced by 1 week (1 W) or 4 W Transverse Aortic Constriction (TAC). To reveal how gene regulation in hypertrophy is related to that in development and to achieve robustness in our analysis, we collected a set of publicly available microarray datasets corresponding to murine heart development at embryonic and postnatal stages, and short- and long-term LVH (Figure 1A and Table S1). Overall, our LVH data included three datasets for 1 W TAC, named LVH1, LVH2, and LVH3, one dataset for 4 W TAC (LVH4), and one dataset for 12 W TAC (LVH5). All TAC samples were compared with sham samples in the same dataset. For embryonic development (ED) of the heart, we divided data into two phases. The first phase, named ED1, was based on comparison of embryonic day (E) 13.5 + E14.5 with E10.5 + E11.5; and the second phase, named ED2, was based on comparison of E16.5 + E18.5 with E13.5 + E14.5. We also obtained a dataset for comparison of embryonic heart at E17 with adult heart, named EA, which can reflect postnatal development. In addition, we included a rat dataset, from which we compared gene expression for postnatal day (P) 20 vs. P1 and P49 vs. P20, named PD1 and PD2, respectively. Expression changes were standardized to make data from heterogeneous sources more comparable (Figure S1).

Using hierarchical clustering, we found, as expected, that development samples were separated from hypertrophy samples, and embryonic development (ED1 and ED2) samples were separated from postnatal development (PD1, PD2 and EA) ones (Figure 1B). Interestingly, gene expression differences between late embryonic stage and adult (EA) in mouse were more similar to those in postnatal development of rat (PD) than to those in embryonic development of mouse (ED), indicating that gene expression changes in rat and mouse at similar developmental stages are well correlated despite different species and array platforms. This result also supports the suitability of using the rat dataset in our analysis.

Among the hypertrophy samples, 1 W TAC samples (LVH1–3) were clustered together and were separated from 4 W and 12 W TAC samples despite that the three 1 W TAC datasets were from different labs and platforms, indicating that genes are distinctly regulated in short- and long-term hypertrophy conditions. As evidenced by the overall clustering pattern (Figure 1B) and Pearson Correlation values (Figure S2), gene expression changes in hypertrophy showed a general negative correlation with those in development, particularly with late embryonic development (ED2) and early postnatal development (PD1). We then identified genes that were significantly regulated in hypertrophy and development. As shown in Figure 1C, more genes were oppositely regulated in hypertrophy vs. development than consistently regulated (261 vs. 89, see Table S3 for the full lists), further supporting the overall inverse correlation between hypertrophy and development (*P*<2.2×10^−16^, Fisher's exact test). In addition, a greater number of genes were found to be regulated in development or hypertrophy only.

We next analyzed Gene Ontology (GO) terms in Biological Process (BP) and Cellular Component (CC) categories for the genes regulated in hypertrophy and development (Figure 1D). Using a new method based on comparison of regulation profiles of different sets of genes (Figure S3 and Text S1 for detail), we found that genes with functions in immune response, extracellular matrix (ECM), cell cycle, and cell morphology tended to be upregulated in hypertrophy, whereas those with functions in energy generation, mitochondrion, and peroxisome tended to be downregulated. Some gene pathways were consistently regulated in hypertrophy and development (Figure 1D), for example, immune response; some pathways were oppositely regulated, such as mitochondrion and cell cycle. The relations were also discernable using gene density plots which showed how genes were distributed with respect to regulation in hypertrophy or development (Figure S4).

Taken together, our result systematically defined genes and pathways regulated in hypertrophy and divided them into development-related and nonrelated groups. The former group corresponds to the “fetal gene program”, which is presumably attributed to reversal of the mechanisms involved in development during hypertrophy.

### Regulation of mRNA isoform expression in cardiac hypertrophy

**Figure 2:**
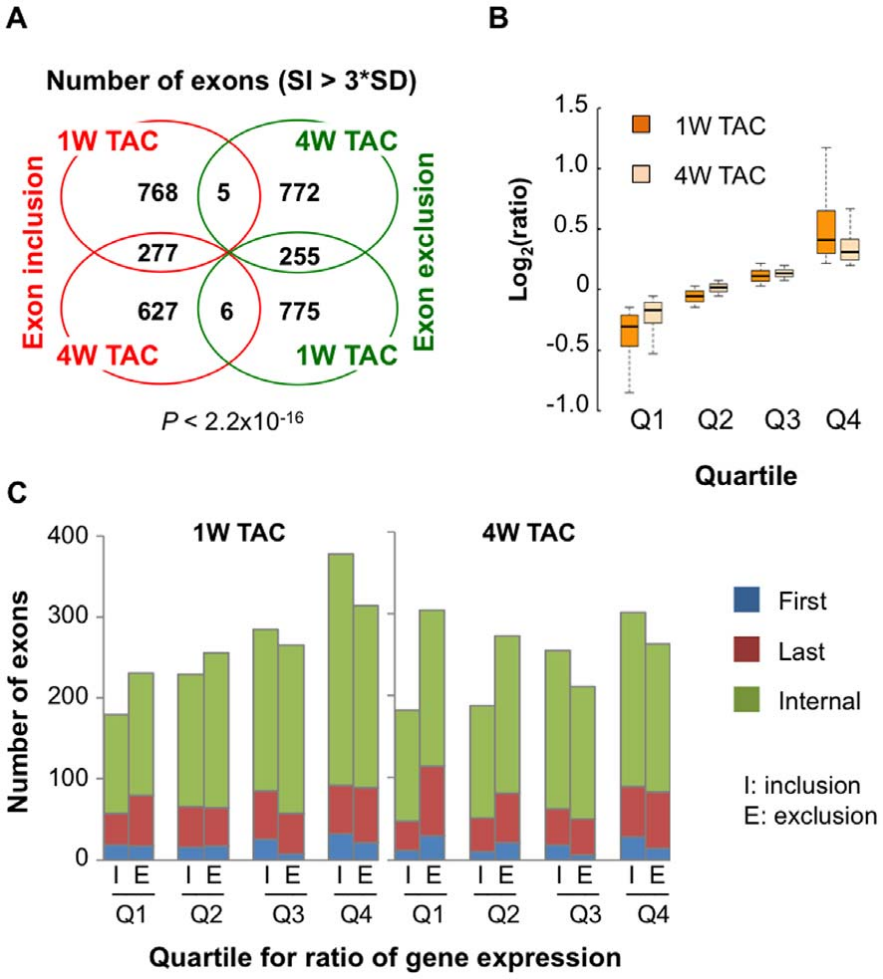
Global analysis of mRNA isoform regulation in cardiac hypertrophy. (**A**) Venn diagram of exon-level regulation in 1 W and 4 W TAC samples (LVH3 and LVH4). Significantly regulated exons are those with splicing index (SI) >3*standard deviation (SD) of all exons. (**B**) Gene expression changes in 1 W and 4 W TAC samples. Genes were divided into four quartiles based on log_2_(ratio). (**C**) Distribution of regulated exons with respect to location and gene expression. Exons were divided into four groups based on gene expression changes as shown in (B), and were further classified into first, internal, and last exons. ‘I’, inclusion; ‘E’, exclusion.

Alternative usage of exonic regions leads to mRNA isoforms encoding different proteins and/or UTRs. To understand how mRNA isoform expression is regulated during hypertrophy, we analyzed exon-level probe intensity changes using our exon array data for 1 W and 4 W TAC and Sham samples. Using the splicing index method, which examines exon usage by normalization to gene expression, and the COSIE method for correction of probe hybridization bias (see Materials and Methods for detail), we identified 2,086 and 1,942 exons with significant change of usage (>3*standard deviation) in 1 W and 4 W TAC samples, respectively (Figure 2A). While a significant fraction of regulated exons (about 14–16% in each sample) were commonly regulated in both 1 W and 4 W TAC samples, suggesting continuous regulation in the course of hypertrophy (*P*<2.2×10^−16^, Fisher's exact test), a large number of stage-specific regulated events were also identified.

Interestingly, we found a correlation between change of gene expression and regulation of internal exon usage in the 1 W TAC samples (Figures 2B and 2C), i.e., genes upregulated at the expression level were more likely to be regulated at the splicing level as well, for both inclusion and exclusion of internal exons. This trend was not discernable in 4 W TAC samples or for 5′ or 3′ terminal exons (Figure 2B). This result suggests that change of gene expression and regulation of splicing variants are coupled at the early stage of hypertrophy (see Discussion).

We next examined GO terms for genes with significant regulation of alternative usage of exons in hypertrophy. While genes with regulated first and/or last exons did not appear to be biased to any GO terms, those with regulated internal exons were found to be significantly associated with a set of GO terms (Table 1). With respect to BP, ‘cell adhesion’, ‘cell cycle phase’, ‘sensory perception of mechanical stimulus’, and ‘microtubule-based process’ were significant for genes with regulated internal exons in 1 W TAC, and ‘ion transport’ and ‘chromosome organization’ were significant for those with regulated internal exons in 4 W TAC. With respect to CC, ‘cytoskeletal part’ was significant for both 1 W and 4 W TAC conditions, whereas ‘proteinaceous extracellular matrix’ was associated with 1 W TAC only. Since many of these GO terms were also associated with regulation of gene expression level (Figure 2), this result further indicates that there is a connection between transcriptional control and splicing regulation during hypertrophy. In addition, this result suggests that alternative splicing (AS) of internal exons can play an important role in remodeling of the heart under pressure overload.

**Table 1:** Significant GO terms for genes with regulated AS.

GO ID, Name	*P* (1W TAC)	*P* (4W TAC)
**Biological Process**		
GO:0007155, cell adhesion	3.6E-06	5.7E-03
GO:0022403, cell cycle phase	7.8E-06	5.1E-03
GO:0050954, sensory perception of mechanical stimulus	1.2E-05	6.2E-02
GO:0007017, microtubule-based process	9.0E-05	4.8E-04
GO:0006811, ion transport	7.4E-02	9.0E-05
GO:0051276, chromosome organization	2.3E-02	9.8E-05
**Cellular Component**		
GO:0044430, cytoskeletal part	3.7E-07	6.1E-05
GO:0005578, proteinaceous extracellular matrix	1.3E-06	2.1E-02
GO:0000775, chromosome, centromeric region	1.4E-04	3.0E-02
GO:0005694, chromosome	1.5E-04	4.9E-04
GO:0042995, cell projection	2.2E-04	6.1E-02

Fisher's exact test was used for genes with significant AS (internal exon) regulation, as shown in Figure 2. Significant GO terms (*P*<0.05 after Benjamini-Hochberg adjustment) in 1 W and/or 4 W TAC are shown, and are sorted by the p-value of 1 W or 4 W TAC, whichever is more significant. Redundant GO terms (sharing more than 50% of associated genes with another GO term having a more significant p-value) were eliminated.

### Correlation of mRNA isoform expression between cardiac hypertrophy and development

**Figure 3:**
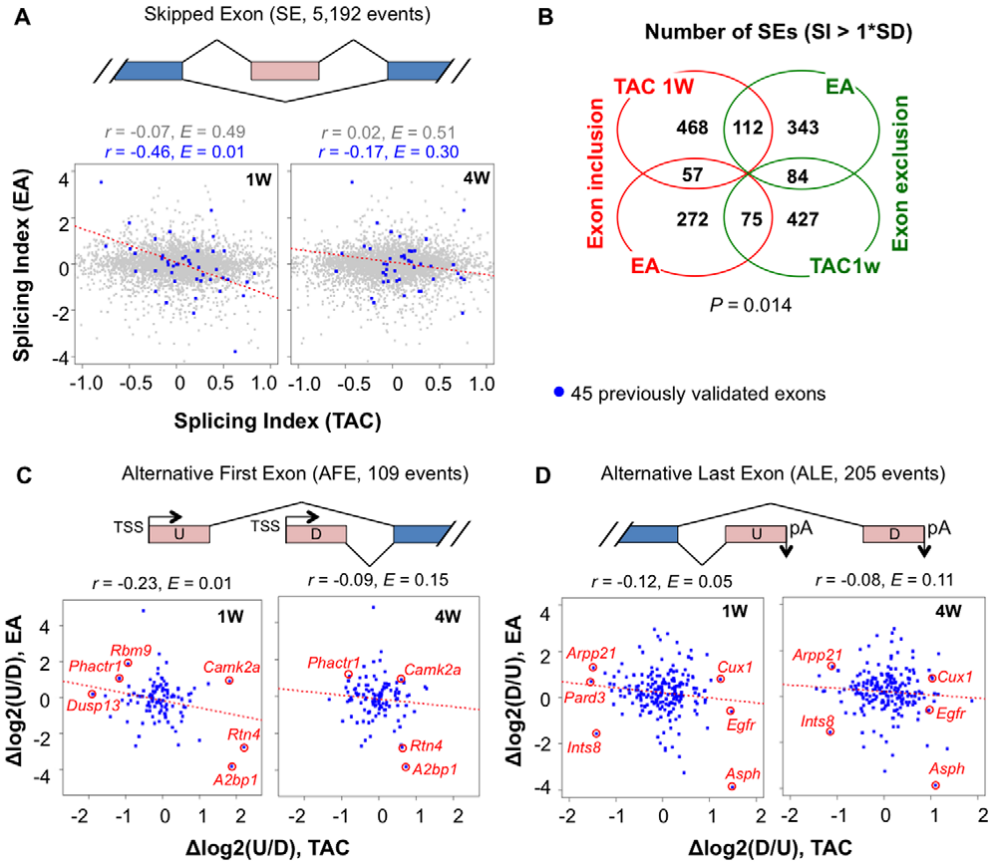
Comparison of mRNA isoform regulation in cardiac hypertrophy and development. (**A**) Comparison of SI for skipped exons (SE). Skipped exons were defined by cDNAs/ESTs. Grey dots are all expressed SE events, and blue dots are the ones that are developmentally regulated and experimentally validated in a previous study [25]. The dotted red line is based on linear regression of blue dots. Pearson correlation coefficient was calculated for each plot, and its *E* value was estimated based on random sampling of the same number of exons (see Materials and Methods for detail). (**B**) Venn diagram showing the highly regulated SEs in hypertrophy and development. Highly regulated SEs were selected using 1*SD of SI. (**C**) and (**D**) Regulation of alternative first and last exons. U and D are upstream and downstream terminal exons, respectively. Alternative terminal exons were analyzed using difference in log_2_(U/D) or log_2_(D/U), where U/D or D/U are ratios of probe set intensities between U and D.

We next asked how the isoform regulation in hypertrophy was related to that in development. To this end, we compared exon usage changes in hypertrophy using our exon array data and those in development using the splicing microarray data (EA) [25]. We focused on three types of isoform changes, namely skipping of internal exon (SE), alternative first exon (AFE), and alternative last exon (ALE) (Figure 3).

For all the 5,192 exons that can be involved in SE, there was no significant correlation between hypertrophy and development, based on Pearson correlation (*r* = −0.07 for 1 W TAC and *r* = 0.02 for 4 W TAC), or on comparison with randomly selected exons (expected value or *E* = 0.49 for 1 W TAC and *E* = 0.51 for 4 W TAC, see Materials and Methods for details of the analysis method). However, when we focused on a set of SE events that were previously validated for regulation in development (Table S4) [25], we detected a significant inverse correlation for 1 W TAC vs. EA (*r* = −0.46, *E* = 0.01) but not for 4 W TAC vs. EA (*r* = −0.17, *E* = 0.30), indicating a set of splicing events are both regulated in hypertrophy and development but in opposite directions. To further explore this, we used a Venn diagram to examine highly regulated SE events in hypertrophy and development. Indeed, more SE events were inversely regulated than consistently regulated (187 vs. 141, *P* = 0.014, Fisher's exact test) (Figure 3B).

For the 109 AFE events analyzed, an inverse correlation between development and hypertrophy was clearly discernable (Figure 3C), with 1 W TAC vs. EA (*r* = −0.23, *E* = 0.01) being more significant than 4 W TAC vs. EA (*r* = −0.09, *E* = 0.15). Several AFE events were highly regulated in LVH and EA with opposite directions, such as *A2bp1*, *Rtn4*, *Rbm9*, and *Phactr1*, but some appeared to be regulated in LVH only, such as *Camk2a* and *Dusp13*. Like AFE events, inverse correlation between development and hypertrophy could also be seen for the 205 ALE events (Figure 3D), and, again, 1 W TAC vs. EA was more significantly correlated (*r* = −0.12, *E* = 0.05) than 4 W TAC vs. EA (*r* = −0.08, *E* = 0.11). Several ALE events were highly regulated in LVH and EA with opposite directions, such as *Asph*, *Egfr*, *Pard3*, *Arpp21*, and some were regulated in LVH only, such as *Cux1*.

Taken together, our result indicates that cardiac hypertrophy involves widespread mRNA isoform changes, and some of the events are oppositely regulated in development, especially for those regulated at the early stage of hypertrophy. These mRNA isoform changes can significantly impact expression of protein isoforms, such as changing protein domains, which are important for adaptation of heart functions under stress overload (See Tables S5, S6, and S7 for top most significantly regulated SE, AFE, and ALE cases in hypertrophy).

### Modulation of expression of Fox family genes may play a role in AS regulation in cardiac hypertrophy

**Figure 4:**
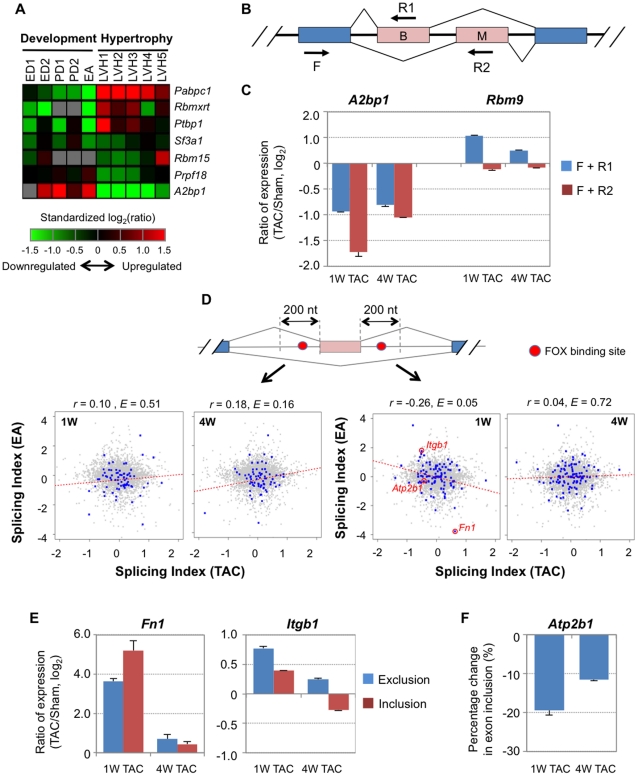
Regulation of splicing factors and Fox target exons in cardiac hypertrophy. (**A**) Gene expression analysis of splicing factors. Splicing factors are those reported in [38], [39]. Selected genes had fold change >1.5 and *P*<0.05 (T-test) in at least one of three 1 W TAC samples and had the same direction of regulation across samples. Genes are shown in a heatmap with color representing standardized log_2_(ratio) based on the color scale shown at the bottom. Grey indicates no detectable expression or not present on microarray. (**B**) and (**C**) Regulation of tissue-specific exons in *A2bp1* and *Rbm9*. ‘B’ and ‘M’ represent brain- and muscle-specific exons, respectively, which are expressed in a mutually exclusive manner. Constitutive exons are in blue and alternative ones are in red. Arrows indicate forward (F) and reverse (R) primers for qRT-PCR. Expression changes are based on comparison with Sham samples. Error bars are standard error of the mean (SEM). (**D**) Splicing regulation for skipped exons with the Fox binding site (TGCATG) located in the upstream or downstream intronic regions adjacent to the exon. Only conserved Fox binding sites were used (see Materials and Methods for detail). Pearson Correlation coefficient and its *E* value are shown for each graph. (**E–F**) Experimental validation of skipped exons with Fox binding sites in the downstream intronic region. See Figure S5 for their exon structures. Exon 25 of *Fn1* and exon 17 of *Itgb1* were analyzed by qRT-PCR and exon 21 of *Atp2b1* was examined by semi-quantitative PCR.

The widespread regulation of AS isoforms prompted us to examine expression of splicing factors. To this end, we examined expression changes of genes that were annotated with splicing activities or previously reported to play roles in AS (193 in total)[38], [39]. A number of genes were found to be highly regulated at the mRNA level during hypertrophy, some of which were oppositely regulated in hypertrophy vs. development. Seven genes that were found to be consistently regulated in all 1 W TAC data sets are shown in Figure 4A. We found that *Pabpc1*, *Rbmxrt*, and *Ptbp1* were upregulated in hypertrophy but downregulated in development, whereas *A2bp1* showed the opposite trend. Notably, *Rbmxrt*, *Ptbp1*, and *A2bp1* all encode proteins (hnRNP G, PTB, and Fox-1, respectively) that have been shown to regulate AS [40], [41], [42].

Since *A2bp1* is highly expressed in the heart [43] and previous studies implicated its role in heart development [25], we focused on its expression regulation and impact on AS in hypertrophy. *A2bp1* belongs to the gene family encoding Fox proteins, which also includes *Rbm9* (Fox-2) and *D11Bwg0517e* (Fox-3). The Fox proteins all contain an RRM RNA binding motif and Fox-1 and Fox-2 have been shown to have high binding specificity to the consensus sequence UGCAUG [44], [45]. Both *A2bp1* and *Rbm9* contain several exons that are differentially used in a tissue-specific manner, including alternative usage of the first exon [43], [46], which was also observed in our study (see above). In addition, exons 16 and 17 of *A2bp1* and their corresponding exons in *Rbm9* (exons 11 and 12) are mutually exclusive exons, which have been found to be highly used in brain and muscle tissues, respectively (Figures 4B and S5). Our exon array data indicated that these two exons were differentially regulated in hypertrophy. To confirm this, we carried out quantitative real-time reverse-transcription PCR (qRT-PCR) using primers targeting two isoforms of *A2bp1* or *Rbm9*. For *A2bp1*, both isoforms were downregulated, but the muscle isoform was more downregulated than the brain isoform, indicating both transcriptional and AS regulation. For *Rbm9*, the overall transcript level did not change significantly but the muscle isoform was mildly downregulated and the brain isoform was mildly upregulated, indicating AS regulation only.

In order to examine the role of Fox proteins in regulation of AS in hypertrophy, we selected skipped exons with conserved Fox binding sites (TGCATG) located either upstream (−200 to −1 nt) or downstream (+1 to +200 nt) of the exon, and analyzed their regulation in hypertrophy vs. development (Figure 4D). Interestingly, exons with Fox binding site(s) in the downstream region were significantly regulated in opposite directions between 1 W TAC and EA (*r* = −0.26, *E* = 0.05, see Table S8 for the exon list). This trend was not discernable for 4 W TAC samples or for exons with Fox binding sites in the upstream region.

We next focused on several skipped exons with Fox binding sites in the downstream intronic region and carried out validation assays, including exons in *Fn1*, *Itgb1*, and *Atp2b1*. *Fn1* and *Itgb1* respectively encode fibronectin and an integrin subunit, two cell adhesion molecules whose interaction modulates contraction of cardiac myocytes [47], [48]. Our microarray data indicated that both genes were upregulated in hypertrophy, and exon 25 of *Fn1* and exon b1D of *Itgb1* had increased inclusion and exclusion in 1 W TAC, respectively (Figure 4D). Using qRT-PCR with primers targeting exon exclusion and inclusion isoforms (Figure S5), we confirmed microarray results both for their gene expression and AS regulation (Figure 4E). We also examined exon 21 in *Atp2b1* (Figure S5), which encodes calcium ion transport ATPase [49], [50]. By semi-quantitative PCR, we also confirmed its regulation in hypertrophy (Figure 4F).

Taken together, our microarray and validation results indicate that Fox-1 protein is likely to play a role in regulation of AS for a set of exons in both 1 W TAC and development, especially when they bind to the downstream intronic region of a skipped exon.

### mRNAs tend to have short 3′UTRs in cardiac hypertrophy

**Figure 5:**
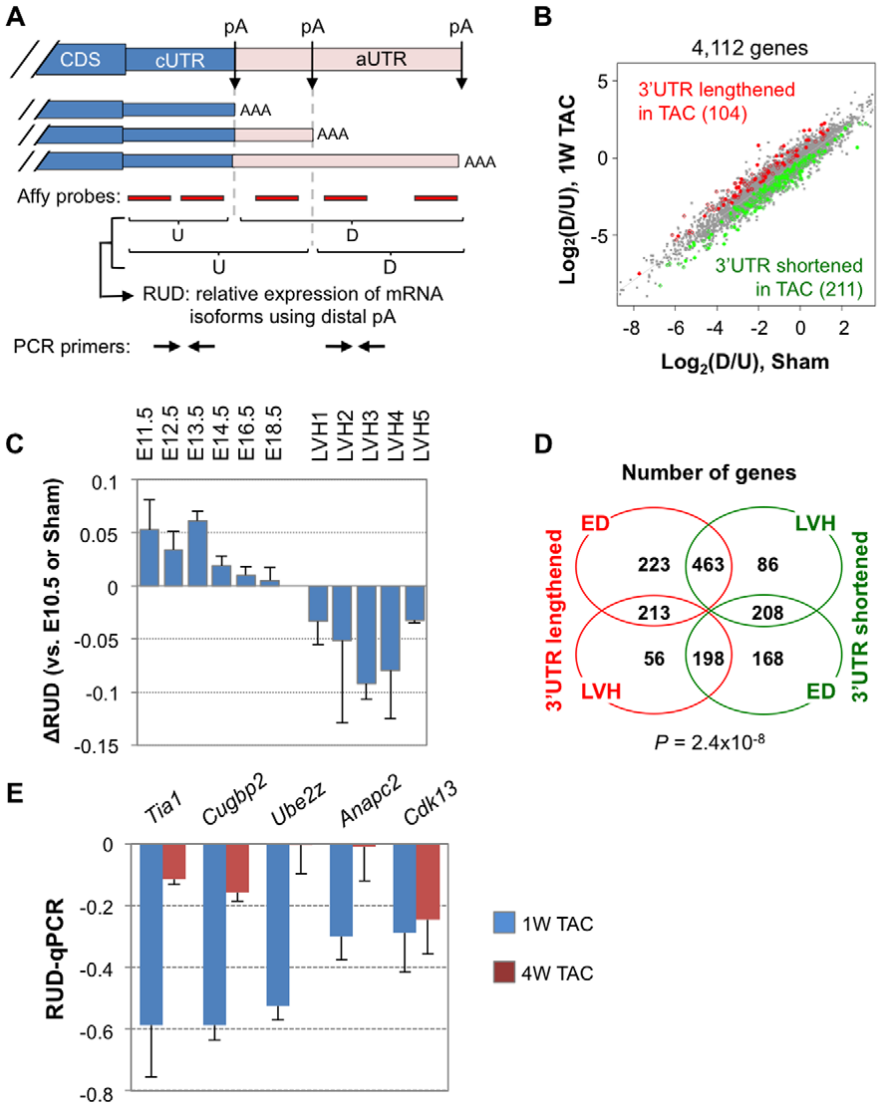
Regulation of 3′UTR isoforms in cardiac hypertrophy and development. (**A**) Schematic of APA and our method to detect 3′UTR length changes using microarray probes. A hypothetical gene contains three polyA sites, resulting in three 3′UTR isoforms. The 3′UTR regions upstream and downstream of the first polyA site are called constitutive and alternative UTRs, or cUTR and aUTR, respectively. Probes mapped to upstream and downstream of each polyA site were used to calculate the RUD score, which reflects the relative expression of 3′UTR isoforms. The RUD score correlates with 3′UTR length. (**B**) Comparison of ratio of probe intensity for the downstream region of polyA site to that of upstream region (D/U) of polyA site between Sham and 1 W TAC (LVH3). A total of 4,112 genes were examined. Genes with significant regulation of 3′UTR length (*P*<0.1, T-test) are colored, with red for genes with 3′UTR lengthened in 1 W TAC and green for those with 3′UTR shortened. (**C**) Global RUD changes in cardiac development and hypertrophy. The median RUD of all genes in each sample was plotted to represent RUD of the sample. All embryonic samples were compared to E10.5, and TAC samples were compared to Sham. Error bars are standard error of the mean (SEM) based on multiple samples. (**D**) Venn diagram showing numbers of genes with UTR lengthened or shortened in development and hypertrophy. We selected genes with consistent regulation in at least 4 out of 6 ED samples and in at least 2 out of 3 LVH samples. Fisher's exact test was used to assess significance of the overlap between development and hypertrophy. (**E**) qRT-PCR analysis of genes with shortened 3′UTRs in TAC. Two sets of PCR primers were designed to target regions upstream and downstream of the first polyA site as shown in (A). The difference in their ratio was used to indicate 3′UTR length changes. PCR primer sequences are listed in Table S2, and gene structures are shown in Figure S6.

Regulation of 3′UTRs by alternative cleavage and polyadenylation (APA) has recently been found to be associated with cell proliferation, oncogenic transformation, and development [34], [35], [36]. To examine how 3′UTRs are regulated in hypertrophy, we took advantage of the fact that a large number of Affymetrix GeneChip probes hybridize to constitutive 3′UTRs (cUTRs) and alternative 3′UTRs (aUTRs) (Figure 5A). cUTRs and aUTRs are defined by polyA sites located in 3′UTRs. We used a score, named relative expression of mRNA isoforms using distal polyA sites (RUD), to indicate the 3′UTR length as illustrated in Figure 5A. RUD was based on comparison of probe intensities for the upstream and downstream regions of a polyA site; a higher score indicates relatively higher abundance of mRNA isoforms resulting from usage of promoter-distal polyA sites, and thus longer 3′UTRs.

As shown in Figure 5B, more genes had higher relative abundance of short 3′UTR isoforms in 1 W TAC as compared to Sham (211 vs. 104), suggesting genes tend to express short 3′UTR isoforms in hypertrophy. Using median RUD score of surveyed genes in each sample, we found 3′UTR shortening took place in all TAC samples (Figure 5C). However, no significant GO terms were found to be associated with genes with shortened 3′UTRs (data not shown), suggesting that 3′UTR isoform regulation is a general feature of hypertrophy affecting all genes.

We next compared 3′UTR regulation in hypertrophy vs. in development. As shown in Figure 5C, compared to E10.5, embryonic development of the heart generally involved more expression of long 3′UTR isoforms, consistent with our previous findings [34]. A significant number of genes were regulated in opposite directions in embryonic development vs. hypertrophy with respect to 3′UTR length regulation (*P* = 2.4×10^−8^, Fisher's exact test, Figure 5D), further indicating an inverse correlation of APA isoform regulation between development and hypertrophy.

To validate our microarray results, we selected a number of genes with 3′UTR length changes, including *Tia1*, *Cugbp2*, *Ube2z*, *Anapc2*, and *Cdk1*. *Tia1* and *Cugbp2* encode RNA binding proteins that play roles in various aspects of post-transcriptional regulation [51], [52]; *Anapc2*, and *Cdk13* encode proteins involved in cell cycle control; and *Ube2z* is involved in the ubiquitin-proteasome pathway [53], [54], [55]. Using qRT-PCR with primer sets targeting upstream and downstream regions of the first polyA site in 3′UTR (Figure 5A and S6), we confirmed 3′UTR shortening in 1 W TAC in all cases. However, this trend was much less obvious in 4 W TAC except for *Cdk13*. Taken together, our result indicates that short 3′UTR isoforms are generally more expressed during hypertrophy, particularly at the early stage. Since mRNAs with short 3′UTRs are generally more stable than long 3′UTR isoforms due to evasion of destabilizing elements, this mechanism could lead to stabilization of mRNAs in hypertrophy, resulting in more protein production.

### miRNA target genes are globally de-repressed in cardiac hypertrophy

**Figure 6:**
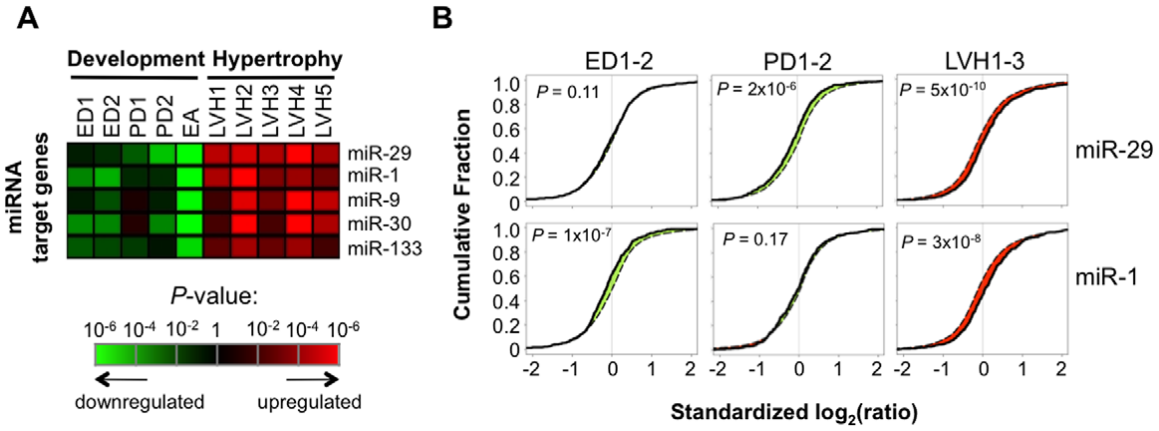
Analysis of expression of miRNA target genes. (**A**) Heatmap of miRNAs whose target genes were significantly regulated in hypertrophy (FDR<0.05). The color represents p-value which was calculated by comparison of CDF curves (see Materials and Methods for detail). The p-values for development datasets were also shown for comparison. miRNAs are sorted by their significance in 1 W TAC. (**B**) Expression changes of the genes targeted by miR-29 and miR-1. CDF curves of standardized log_2_(ratio) values are shown for ED, PD, and 1 W TAC samples. ED and PD are based on averaged values across ED and PD samples, respectively. 1 W TAC is based on averaged values of three samples (LVH1–3). P-values indicating difference between CDFs of miRNA target genes and other genes were derived from the Kolmogorov–Smirnov test.

One of the mechanisms regulating mRNA expression via 3′UTRs is miRNA-mediated destabilization of transcripts. To examine how miRNA target genes are regulated in hypertrophy, we compared the regulation profile of miRNA target genes as predicted by TargetScan [56] with that of other genes for every known miRNA family. As shown in Figure 6A, mRNA targets of several miRNA families were found to be significantly upregulated in hypertrophy (false discovery rate (FDR) <0.05), including those targeted by miR-29, miR-1, miR-9, miR-30, and miR-133. Notably, except for miR-9, these miRNAs are all highly abundant in the heart [17], indicating that our result has the potential to be physiologically relevant. Interestingly, no miRNAs were predicted to have an enhanced function in hypertrophy, i.e. more repression of target mRNA expression, suggesting that miRNA activity is generally inhibited in cardiac hypertrophy. We also compared hypertrophy with development with respect to these miRNAs. As shown in Figure 6A, mRNA targets of all these miRNAs were downregulated in development, but their significance depended on the developmental stage. For example, miR-29 target genes were more downregulated in postnatal development, whereas miR-1 target genes were mainly downregulated in embryonic development (Figure 6B). This result indicates that miRNAs might play an important role in defining the gene expression program in hypertrophy and contribute to the activation of the ‘fetal gene program’. In addition, combined with our observation that 3′UTRs are generally shortened in cardiac hypertrophy, the miRNA result suggests that inhibition of mRNA stability and/or translation is generally avoided in hypertrophy.

## Discussion

Our comprehensive analysis of microarray data from multiple studies identified genes and pathways significantly and robustly regulated in cardiac hypertrophy. These results, while generally consistent with previous studies [57], [58], [59], [60], systematically refine and extend the current understanding of regulation of transcriptome in cardiac hypertrophy. Comparison of hypertrophy with embryonic and postnatal development enabled us to systematically define the fetal gene program. In addition, our genome-wide exon-level analysis indicates that regulation of mRNA isoform is widespread during cardiac hypertrophy. While some isoform changes are hypertrophy-specific, other events are negatively correlated with those in development, particularly for the events regulated at the early stage of hypertrophy, suggesting activation of a fetal post-transcriptional program in the heart in response to pressure overload.

Our GO analysis identified a number of pathways that are regulated in both development and hypertrophy. For some pathways, regulation in development and in hypertrophy involves the same set of genes, for example genes related to mitochondrion functions. This can be discerned using gene density plots (Figure S4). However, for some other pathways, regulation of a pathway in hypertrophy involves a different set of genes than in development. For example, despite cell cycle genes as a set are upregulated in hypertrophy and downregulated in development, regulation of individual genes is quite different in these two conditions, as indicated in the gene density plot (Figure S4). This is consistent with the notion that development involves cell proliferation whereas hypertrophy involves expansion of cell size. In addition, gene regulation for some pathways is quite complex, particularly genes related to ECM (for example, proteinaceous extracellular matrix). They are globally upregulated in hypertrophy, but in development only a subset of them is downregulated and another subset is upregulated.

We found that genes upregulated at the expression level are also more likely to be regulated at the splicing level in 1 W TAC, suggesting coupling splicing regulation to transcription. This observation is not due to technical reasons, because this coupling is not discernable in 4 W TAC samples, and we corrected splicing index values for hybridization biases related to gene expression changes [61]. Regulation of AS by transcription can be attributable to difference in recruitment of splicing factors to RNA polymerase II (Pol II), known as recruitment coupling, or change in Pol II elongation rate, known as kinetic coupling [62], [63]. On this note, *Cdk9*, which encodes a Pol II elongation factor, has been shown to be regulated in hypertrophy [64]. Further studies are needed to unravel this phenomenon.

Our expression analysis indicates that a number of splicing factors are regulated in hypertrophy, which presumably contributes to global AS regulation. We focused on the gene encoding Fox-1, which, interestingly, is significantly regulated at several levels, including expression, alternative initiation, and AS. While motif analysis did not reveal an enrichment of the Fox binding site near regulated exons (data not shown), we did observe a negative correlation between hypertrophy and development for the skipped exons with the Fox binding site located in downstream of exon (Figure 4D). This result suggests that regulation of Fox-1 may play a role in establishing the fetal splicing program in the hypertrophied heart. Interestingly, PTB, which was previously found to interact with Fox in AS regulation [65], [66], is significantly upregulated at the mRNA level. How these two proteins function and interact with each other in hypertrophy is to be further examined. In addition, given that posttranscriptional and posttranslational regulation of splicing factors is very common, it is highly possible that other splicing factors may also play roles in AS regulation in hypertrophy despite that their mRNA levels do not significantly change as found in this study.

Diversity in 3′ end formation of mRNA has recently been highlighted as an important layer of gene regulation [67], [68], [69]. Previous studies have reported general 3′UTR shortening in proliferation and oncogenic transformation [35], [36]. Here we extend this phenomenon to cardiac hypertrophy. It is possible that mechanisms of 3′UTR regulation in hypertrophy and proliferation are related, because 1) a set of cell cycle genes are upregulated in hypertrophy, and 2) we previously found that pre-mRNA processing genes typically have binding sites for cell cycle-related transcription factors in their promoters [37]. Consistent with this notion, we found that mRNAs of CstF proteins, which have been shown previously to play a role in regulation of APA [70], were upregulated after 3 days of TAC (Figure S7). Interestingly, their upregulation was subdued after 1 week of TAC. Therefore, 3′UTR isoform regulation may be executed at a very early stage of hypertrophy and the effect remains during TAC.

Shortening of 3′UTR can lead to more protein production by avoiding destabilizing cis elements in the 3′UTR, such as miRNA target sites, AREs and GREs. Consistently, despite reports implicating upregulation of some miRNAs in hypertrophy [18], we found most miRNA target genes were upregulated during hypertrophy, indicating a global de-repression of miRNA targeting. These results suggest coordinated mechanisms to stabilize mRNAs in hypertrophy, which can be important for rapid enlargement of heart in response to mechanical stress.

## Materials and Methods

### Exon array of TAC samples

All animal work has been conducted according to a protocol approved by the Institutional Animal Care and Use Committee (IACUC) at UMDNJ-New Jersey Medical School. Protocol number: 07120. Transverse Aortic Constriction (TAC) was conducted as described previously [71]. Briefly, aortic constriction was performed by ligation of the transverse aorta with a 28-gauge needle using a 7-0 braided polyester suture. Sham operation was performed without constricting the aorta. Two mice (C57BL/6) were used for 1 W and 4 W TAC and Sham. Total RNA from left ventricles was extracted using the RNeasy Fibrous Tissue Mini Kit (QIAGEN) and was processed by the Whole Transcript (WT) Sense Target Labeling kit (Affymetrix) for exon array analysis (Affymetrix GeneChip Exon Mouse 1.0 ST Array). Our data is deposited in the GEO database of NCBI (GSE: 24242) and is MIAME compliant. See Table S1 for details on the public datasets used in this study.

### Microarray data analysis

Microarray data normalization was carried out using the robust multi-chip analysis (RMA) method. To determine whether a gene was expressed, we used the MAS 5.0 method for 3′ arrays and the detection above background (DABG) method for exon arrays. Only genes with detectable expression in >50% of samples in a comparing group were used for further analysis. For the splicing microarray, we mapped probes to exons of gene models based on cDNAs/ESTs, as described previously [72]. Gene expression was calculated using constitutively expressed exons only.

### Gene set analysis

Comparison of gene sets for expression changes was based on the cumulative distribution function (CDF)(See Text S1 for detail). Gene Ontology information was obtained from the NCBI Gene database. miRNA target sites were obtained from the TargetScan database (v5.1) [73]. To evaluate FDR for our selection at a given p-value, three datasets (LVH1, LVH2, LVH3) were randomized (sample-wise), and the number of falsely identified entries at a given p-value was calculated.

### mRNA isoform analysis

Exon usage was analyzed using the splicing index (SI) method, which is based on expression change of a given exon vs. expression change of its corresponding gene. Since different probes can respond to transcript changes differently, SI values were adjusted by the Corrected Splicing Index for Exon Arrays (COSIE) method [61]. Classification of first, last, and internal exons was based on the RefSeq database. Skipped exons were identified using cDNAs/ESTs [72]. Fox binding sites were analyzed by a method similar to the one previously reported [74]. Expected value (*E*) for Pearson Correlation for a given set of exons corresponds to the fraction of times (out of 1,000 times) that a randomly selected set including the same number of exons has a better correlation. Analysis of Fox binding sites was based on the method previously developed [74]. Information about alternative polyA sites was retrieved from PolyA_DB [72]. Analysis of APA isoforms was carried out by the RUD method, as previously described [34].

### Experimental validation

To validate microarray data, we used real-time reverse transcription PCR (qRT-PCR) or semi-quantitative RT-PCR (see Table S2 for primers used in this study). PCR was carried out using the SYBR Green method with *Gapdh* mRNA as an internal control. For semi-quantitative RT-PCR, primer sets targeting constitutive exons flanking a skipped exon were used and PCR products were analyzed by ImageJ (http://rsbweb.nih.gov/ij/index.html).

## Supporting Information

Figure S1 **Standardization of log_2_(ratio) makes data more comparable across datasets.** **(A)** Distribution of gene expression changes (log_2_(ratio)) of all datasets used in this study. Development samples are shown in green and hypertrophy samples are shown in orange. **(B)** Distribution of gene expression changes after standardization.
(TIF)

Figure S2 **Correlation between hypertrophy and development samples.** Pair-wise Pearson Correlation coefficients between samples are shown in a heatmap according to the color scale shown in the graph.
(TIF)

Figure S3 **Analysis gene sets using expression change profiles.** **(A)** An example of gene set analysis using cumulative distribution function (CDF). Genes annotated with “proteinaceous extracellular matrix” form a gene set. The black line is CDF curve for genes in the set, and the dotted line is CDF curve for other genes on the microarray. The difference between two CDF curves, or ΔCDF, is indicated by color, with green for negative values and red for positive ones. ΔCDF was used to examine regulation of a gene set, which yielded two p-values, one for positive values or ΔCDF(+), representing upregulation, and one for negative values or ΔCDF(-), representing downregulation (see Supplementary Materials and Methods, Text S1, for detail). **(B)** Log_2_(gene set size) vs. log_2_(SD of ΔCDF). A linear regression line is shown in red, and its *R* ^2^ is indicated in the graph. The data is derived from randomly sampled genes (10,000 times for each gene set size). **(C)** Distribution of standardized ΔCDF follows the exponential distribution with rate (λ)  = 1.1. The thick black line is distribution of standardized ΔCDF and the dotted red line is exponential distribution. The two distributions are not significantly different when the values are >2 (*P* = 0.29, Kolmogorov-Smirnov test).
(TIF)

Figure S4 **Gene density plots for several significant GO terms.** (A) An example of the gene density plot showing correlation between hypertrophy and development. X-axis is log2(ratio) in development, and y-axis is averaged log2(ratio) in three 1W TAC samples. The horizontal and vertical white lines in each plot mark the point with log2(ratio)  = 0. **(B)** Gene density plots for top 5 BP (top) and CC (bottom) GO terms. See Supplementary Materials and Methods (Text S1) for details of the method.
(TIF)

Figure S5 **Information about validated genes with AS regulation in hypertrophy.** For each gene, a gene structure image is derived from UCSC genome browser. Conservation based on 17 vertebrate species is indicated in the magnified image. A schematic indicating splicing pattern is shown at the bottom. Exon numbers are indicated. Constitutive exons are shown in blue and alternative ones are in red. PCR primers are shown as arrows.
(TIF)

Figure S6 **Information about validated genes with APA regulation in hypertrophy.** For each gene, a gene structure image is derived from UCSC genome browser. Conservation based on 17 vertebrate species is indicated in the magnified image. PolyA site is shown as pA; PCR primers are shown as arrows.
(TIF)

Figure S7 **Regulation of** ***Cstf*** **genes in hypertrophy.** qRT-PCR data are shown for *Cstf1*, *Cstf2*, and *Cstf3* in Sham, 3 day(D) TAC, and 1W TAC. Error bars are standard deviation based on 3 mice.
(TIF)

Text S1 **Supplementary materials and methods and references for supporting information.**
(DOCX)

Table S1 **Datasets used in this study.**
(DOCX)

Table S2 **Primers used in this study.**
(DOCX)

Table S3 **Genes significantly regulated in hypertrophy and/or development.**
(DOCX)

Table S4 **Forty-five developmentally regulated skipped exons.**
(DOCX)

Table S5 **Top 50 most significantly regulated exon skipping events in hypertrophy.**
(DOCX)

Table S6 **Top 5 regulated AFE events in hypertrophy.**
(DOCX)

Table S7 **Top 5 regulated ALE events in hypertrophy.**
(DOCX)

Table S8 **Skipped exons with Fox binding sites in the downstream intronic region.**
(DOCX)
