# Supplementary figures and images for: Comparative Analysis of mRNA Isoform Expression in Cardiac Hypertrophy and Development Reveals Multiple Post-Transcriptional Regulatory Modules
Source: PLoS One. 2011 Jul 22;6(7):e22391. doi: 10.1371/journal.pone.0022391 (PMC3142162; doi:10.1371/journal.pone.0022391)

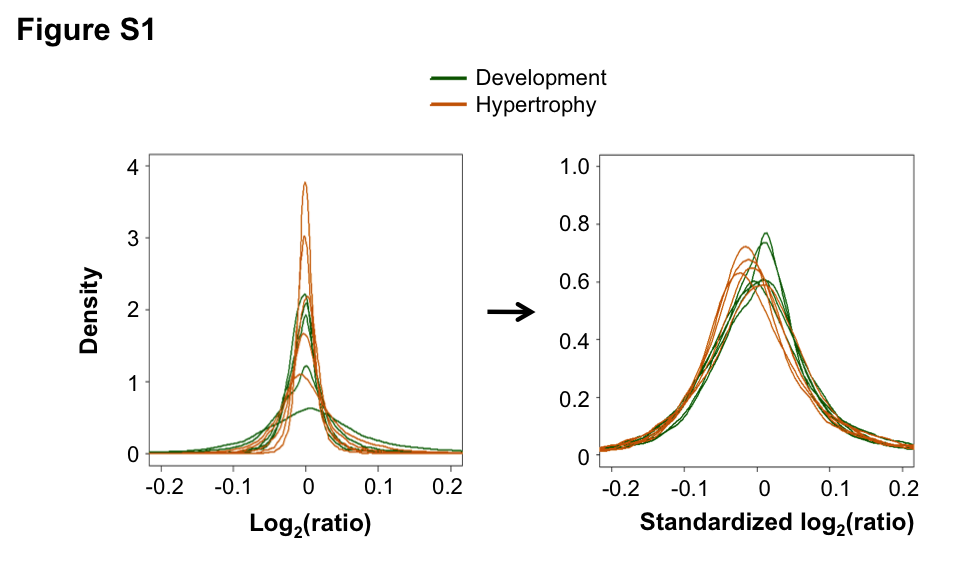

Supplement: Figure S1 — Standardization of log2(ratio) makes data more comparable across datasets. (A) Distribution of gene expression changes (log2(ratio)) of all datasets used in this study. Development samples are shown in green and hypertrophy samples are shown in orange. (B) Distribution of gene expression changes after standardization. (TIF) [file pone.0022391.s001.tif]

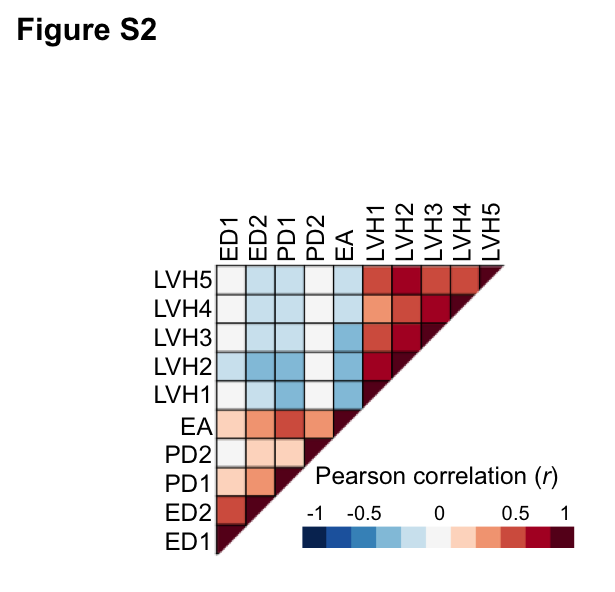

Supplement: Figure S2 — Correlation between hypertrophy and development samples. Pair-wise Pearson Correlation coefficients between samples are shown in a heatmap according to the color scale shown in the graph. (TIF) [file pone.0022391.s002.tif]

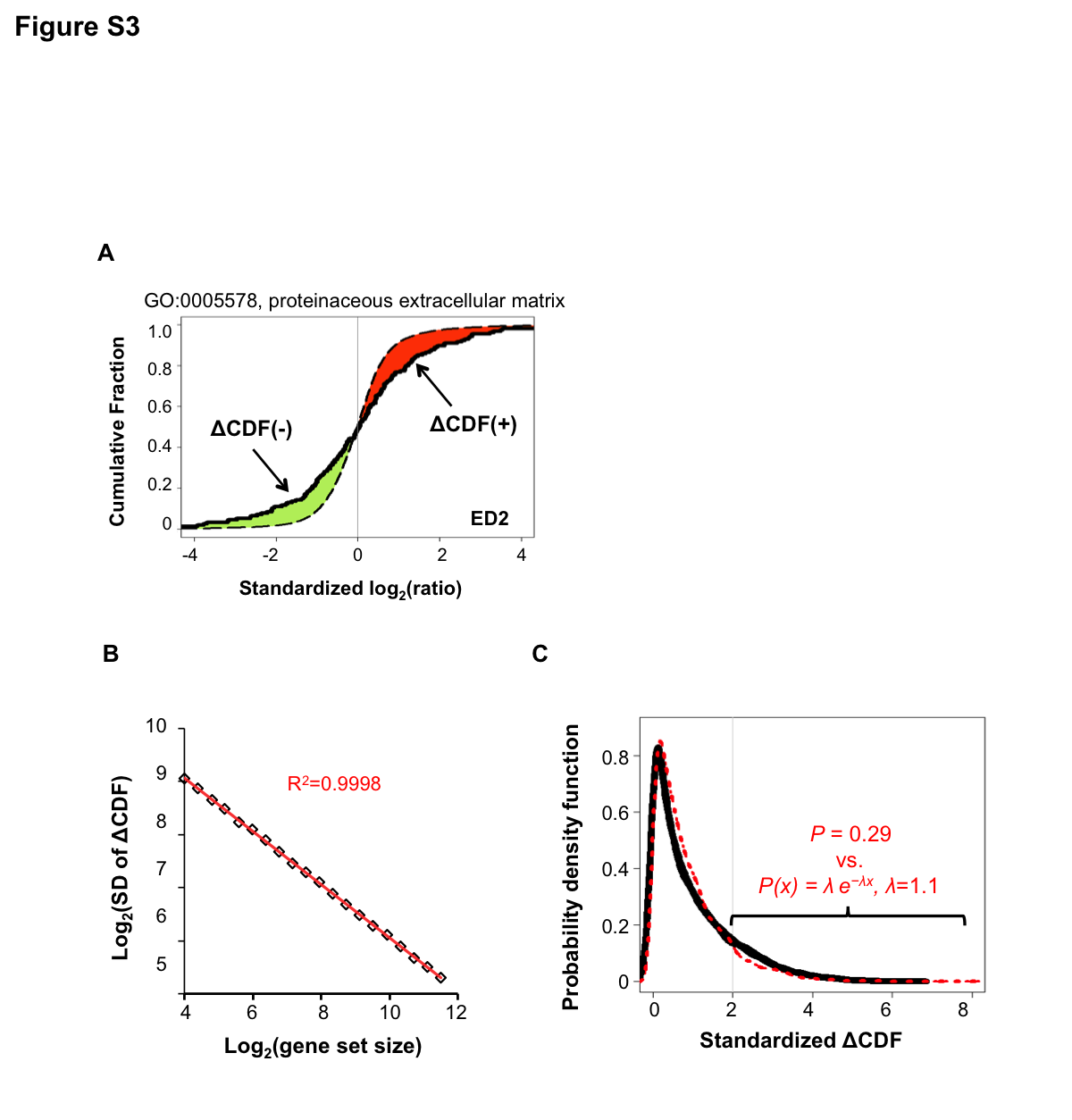

Supplement: Figure S3 — Analysis gene sets using expression change profiles. (A) An example of gene set analysis using cumulative distribution function (CDF). Genes annotated with “proteinaceous extracellular matrix” form a gene set. The black line is CDF curve for genes in the set, and the dotted line is CDF curve for other genes on the microarray. The difference between two CDF curves, or ΔCDF, is indicated by color, with green for negative values and red for positive ones. ΔCDF was used to examine regulation of a gene set, which yielded two p-values, one for positive values or ΔCDF(+), representing upregulation, and one for negative values or ΔCDF(-), representing downregulation (see Supplementary Materials and Methods, Text S1, for detail). (B) Log2(gene set size) vs. log2(SD of ΔCDF). A linear regression line is shown in red, and its R 2 is indicated in the graph. The data is derived from randomly sampled genes (10,000 times for each gene set size). (C) Distribution of standardized ΔCDF follows the exponential distribution with rate (λ) = 1.1. The thick black line is distribution of standardized ΔCDF and the dotted red line is exponential distribution. The two distributions are not significantly different when the values are >2 (P = 0.29, Kolmogorov-Smirnov test). (TIF) [file pone.0022391.s003.tif]

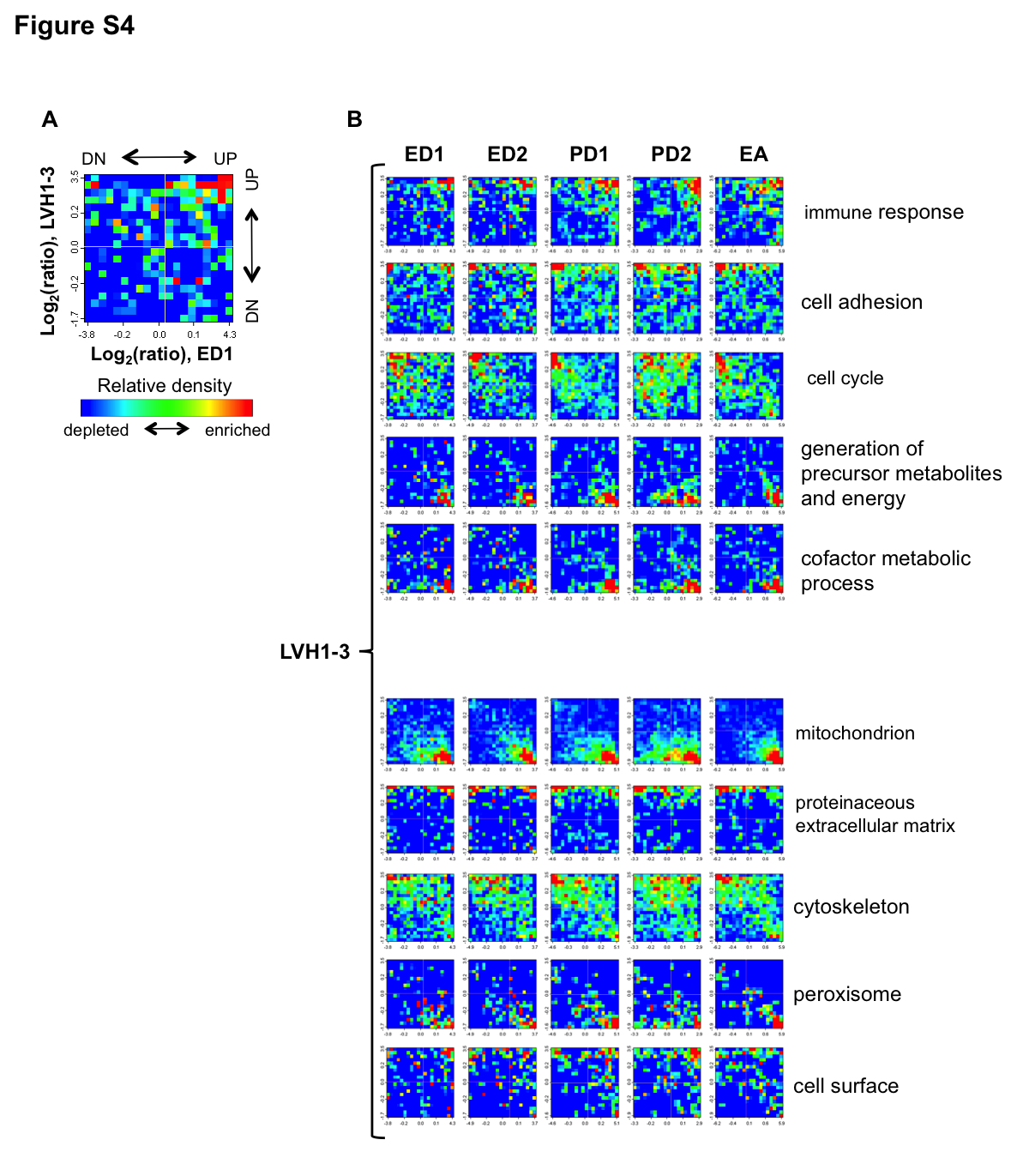

Supplement: Figure S4 — Gene density plots for several significant GO terms. (A) An example of the gene density plot showing correlation between hypertrophy and development. X-axis is log2(ratio) in development, and y-axis is averaged log2(ratio) in three 1W TAC samples. The horizontal and vertical white lines in each plot mark the point with log2(ratio) = 0. (B) Gene density plots for top 5 BP (top) and CC (bottom) GO terms. See Supplementary Materials and Methods (Text S1) for details of the method. (TIF) [file pone.0022391.s004.tif]

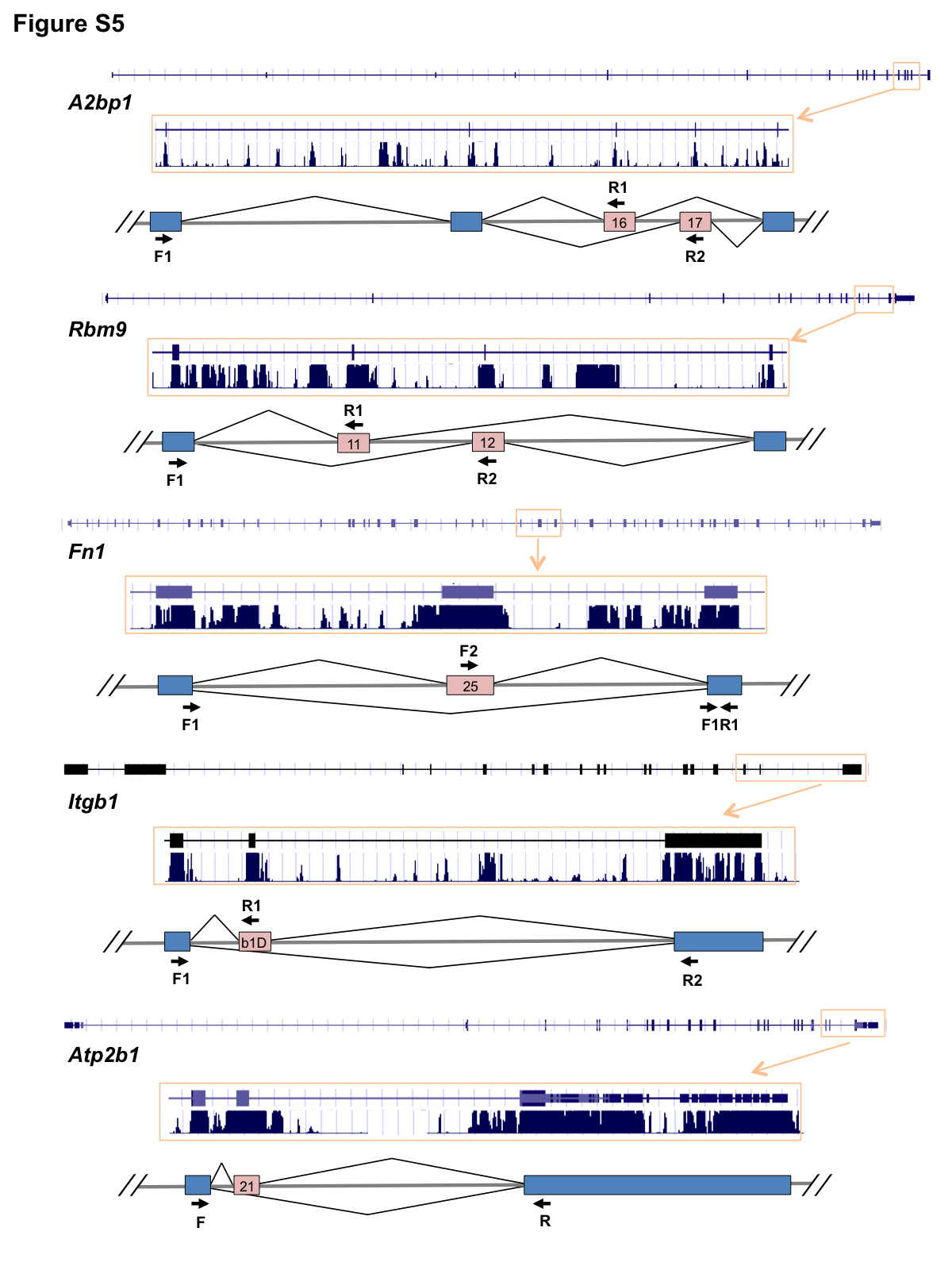

Supplement: Figure S5 — Information about validated genes with AS regulation in hypertrophy. For each gene, a gene structure image is derived from UCSC genome browser. Conservation based on 17 vertebrate species is indicated in the magnified image. A schematic indicating splicing pattern is shown at the bottom. Exon numbers are indicated. Constitutive exons are shown in blue and alternative ones are in red. PCR primers are shown as arrows. (TIF) [file pone.0022391.s005.tif]

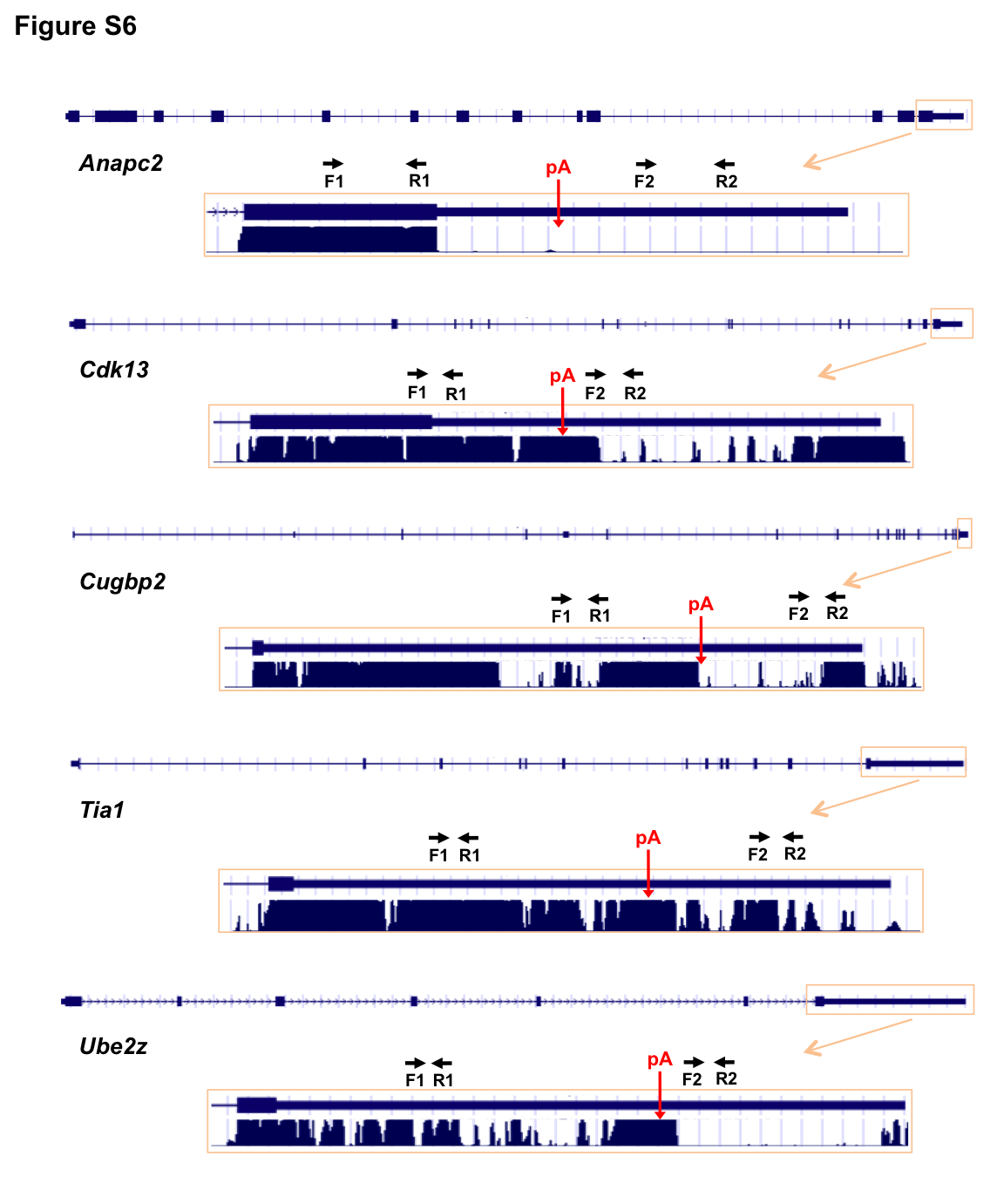

Supplement: Figure S6 — Information about validated genes with APA regulation in hypertrophy. For each gene, a gene structure image is derived from UCSC genome browser. Conservation based on 17 vertebrate species is indicated in the magnified image. PolyA site is shown as pA; PCR primers are shown as arrows. (TIF) [file pone.0022391.s006.tif]

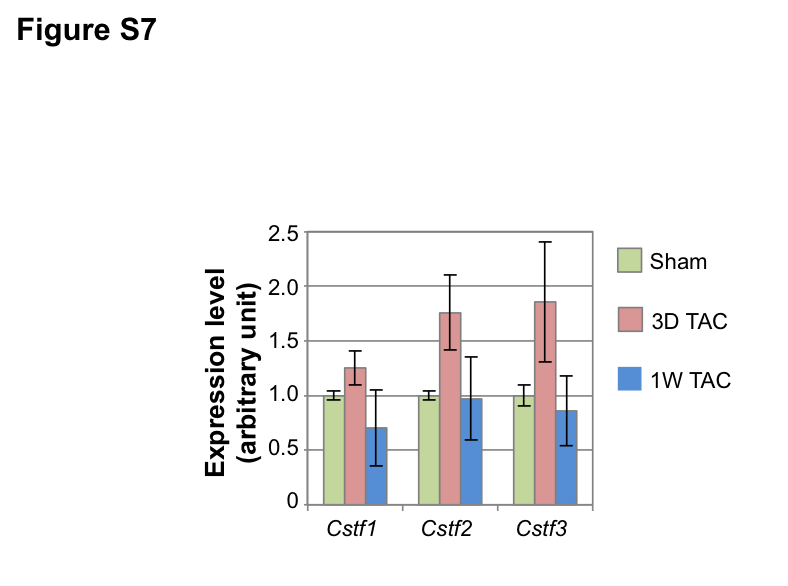

Supplement: Figure S7 — Regulation of Cstf genes in hypertrophy. qRT-PCR data are shown for Cstf1, Cstf2, and Cstf3 in Sham, 3 day(D) TAC, and 1W TAC. Error bars are standard deviation based on 3 mice. (TIF) [file pone.0022391.s007.tif]
