# Supplementary material for: Comparative Analysis of mRNA Isoform Expression in Cardiac Hypertrophy and Development Reveals Multiple Post-Transcriptional Regulatory Modules
Source: PLoS One. 2011 Jul 22;6(7):e22391. doi: 10.1371/journal.pone.0022391 (PMC3142162; doi:10.1371/journal.pone.0022391)
Supplement: Text S1 — Supplementary materials and methods and references for supporting information. (DOCX) [file pone.0022391.s008.docx]

**Text S1**

**Supplementary Materials and Methods**

**Public datasets used in this study**. Public microarray datasets were downloaded from the GEO database of NCBI (<http://www.ncbi.nlm.nih.gov/projects/geo/>) or obtained from the authors (Table S1). The hypertrophy data include 1W TAC (LVH1, GSE5500) and 12W TAC (LVH5, GSE7781) from GEO and LVH2 (1W TAC) obtained from the authors [[1](#_ENREF_1)]. The embryonic development dataset (GSE1479) includes whole heart samples at E10.5 and E11.5, and left ventricle samples at E13.5, E14.5, E16.5, and E18.5. E13.5 + E14.5 vs. E10.5 + E11.5 represents early embryonic development (ED1), and E16.5 + E18.5 vs. E13.5 + E14.5 represents late embryonic development (ED2). The postnatal development dataset (GSE11137) includes rat ventricle samples at day 1, day 20, and day 49 after birth. Rat genes were mapped to homologous mouse genes using the NCBI HomoloGene database. The splicing microarray data for mouse adult vs. E17 was kindly provided by John Castle [[2](#_ENREF_2)].

**Gene set analysis.** Our method to study gene set regulation, including GO analysis and miRNA target gene analysis, is based on comparison of profiles for expression changes using the cumulative distribution function (CDF). First, CDF of log_2_(ratio) of a gene set was compared to that of other genes on the microarray. The difference between the two CDFs, named ΔCDF, were calculated and standardized by the standard deviation (SD) which was derived from random sampling of data (Figure S3A). We found there was a linear correlation between log_2_(SD of ΔCDF) and log_2_(gene set size). Therefore, we made a standard curve to derive SD for any given gene set size, which was constructed by using randomly assembled gene sets of different sizes (Figure S3B). We also found the null distribution of standardized ΔCDF followed an exponential distribution (rate=1.1) when values are >2 (Figure S3C). Therefore, we used the exponential distribution to derive p-values for standardized ΔCDF. Since this method is based on CDFs derived from the same dataset, technical discordance between datasets due to differences in experimental design, array platform, etc., can be reduced and there is no requirement of using arbitrary cutoffs to select genes for analysis. The detailed technical description of this method, comparison with other methods, and its applications will be published elsewhere.

**Gene density map.** Gene density maps were used to compare gene expression changes in two conditions, e.g., hypertrophy vs. development. First, based on expression ratios in each condition, a 20x20 table was constructed with each row and column containing approximately the same number of genes. Each cell has a defined range of expression ratios for each condition. Genes were distributed in the table according to their expression ratios in the two conditions. The number of genes in a cell (observed value) relative to the median of the table (expected value) was calculated and presented in a heatmap using the function kde2d in program R. The color scale used in the heatmap was determined by the 2^nd^- and 98^th^-percentiles of all values.

**References for Supporting Information:**

1. Matsuda T, Zhai P, Maejima Y, Hong C, Gao S, et al. (2008) Distinct roles of GSK-3alpha and GSK-3beta phosphorylation in the heart under pressure overload. Proc Natl Acad Sci U S A 105: 20900-20905.

2. Kalsotra A, Xiao X, Ward AJ, Castle JC, Johnson JM, et al. (2008) A postnatal switch of CELF and MBNL proteins reprograms alternative splicing in the developing heart. Proc Natl Acad Sci U S A 105: 20333-20338.

3. Greaser ML, Warren CM, Esbona K, Guo W, Duan Y, et al. (2008) Mutation that dramatically alters rat titin isoform expression and cardiomyocyte passive tension. Journal of Molecular and Cellular Cardiology 44: 983-991.

4. Bisping E, Ikeda S, Kong SW, Tarnavski O, Bodyak N, et al. (2006) Gata4 is required for maintenance of postnatal cardiac function and protection from pressure overload-induced heart failure. Proc Natl Acad Sci U S A 103: 14471-14476.

5. Matsuda T, Zhai P, Maejima Y, Hong C, Gao S, et al. (2008) Distinct roles of GSK-3{alpha} and GSK-3{beta} phosphorylation in the heart under pressure overload. Proc Natl Acad Sci U S A.

6. Kuba K, Zhang L, Imai Y, Arab S, Chen M, et al. (2007) Impaired heart contractility in Apelin gene-deficient mice associated with aging and pressure overload. Circ Res 101: e32-42.

7. Hoersch S, Andrade-Navarro MA (2010) Periostin shows increased evolutionary plasticity in its alternatively spliced region. BMC Evol Biol 10: 30.

8. Horiuchi K, Amizuka N, Takeshita S, Takamatsu H, Katsuura M, et al. (1999) Identification and characterization of a novel protein, periostin, with restricted expression to periosteum and periodontal ligament and increased expression by transforming growth factor beta. J Bone Miner Res 14: 1239-1249.

9. Khatri JJ, Joyce KM, Brozovich FV, Fisher SA (2001) Role of myosin phosphatase isoforms in cGMP-mediated smooth muscle relaxation. Journal of Biological Chemistry 276: 37250-37257.

10. Wullrich-Schmoll A, Kilimann MW (1996) Structure of the human gene encoding the phosphorylase kinase beta subunit (PHKB). Eur J Biochem 238: 374-380.

11. Dupuis L, Gonzalez de Aguilar JL, di Scala F, Rene F, de Tapia M, et al. (2002) Nogo provides a molecular marker for diagnosis of amyotrophic lateral sclerosis. Neurobiol Dis 10: 358-365.

12. Yan R, Shi Q, Hu X, Zhou X (2006) Reticulon proteins: emerging players in neurodegenerative diseases. Cell Mol Life Sci 63: 877-889.

13. Loyer P, Trembley JH, Grenet JA, Busson A, Corlu A, et al. (2008) Characterization of cyclin L1 and L2 interactions with CDK11 and splicing factors: influence of cyclin L isoforms on splice site selection. J Biol Chem 283: 7721-7732.

14. Schultz DW, Klein ML, Humpert AJ, Luzier CW, Persun V, et al. (2003) Analysis of the ARMD1 locus: evidence that a mutation in HEMICENTIN-1 is associated with age-related macular degeneration in a large family. Hum Mol Genet 12: 3315-3323.

15. Mio T, Yabe T, Arisawa M, Yamada-Okabe H (1998) The eukaryotic UDP-N-acetylglucosamine pyrophosphorylases. Gene cloning, protein expression, and catalytic mechanism. J Biol Chem 273: 14392-14397.

16. Mizushima N, Kuma A, Kobayashi Y, Yamamoto A, Matsubae M, et al. (2003) Mouse Apg16L, a novel WD-repeat protein, targets to the autophagic isolation membrane with the Apg12-Apg5 conjugate. J Cell Sci 116: 1679-1688.

17. Khrebtukova I, Kuklin A, Woychik RP, Michaud EJ (1999) Alternative processing of the human and mouse raly genes(1). Biochim Biophys Acta 1447: 107-112.

18. Lee JA, Tang ZZ, Black DL (2009) An inducible change in Fox-1/A2BP1 splicing modulates the alternative splicing of downstream neuronal target exons. Genes Dev 23: 2284-2293.

19. Bae SC, Ogawa E, Maruyama M, Oka H, Satake M, et al. (1994) PEBP2 alpha B/mouse AML1 consists of multiple isoforms that possess differential transactivation potentials. Mol Cell Biol 14: 3242-3252.

20. Madaule P, Eda M, Watanabe N, Fujisawa K, Matsuoka T, et al. (1998) Role of citron kinase as a target of the small GTPase Rho in cytokinesis. Nature 394: 491-494.

21. Emig D, Salomonis N, Baumbach J, Lengauer T, Conklin BR, et al. (2010) AltAnalyze and DomainGraph: analyzing and visualizing exon expression data. Nucleic Acids Res 38: W755-762.

22. Chen HH, Luche R, Wei B, Tonks NK (2004) Characterization of two distinct dual specificity phosphatases encoded in alternative open reading frames of a single gene located on human chromosome 10q22.2. J Biol Chem 279: 41404-41413.

23. Nakahata S, Kawamoto S (2005) Tissue-dependent isoforms of mammalian Fox-1 homologs are associated with tissue-specific splicing activities. Nucleic Acids Res 33: 2078-2089.

24. Bayer KU, Lohler J, Harbers K (1996) An alternative, nonkinase product of the brain-specifically expressed Ca2+/calmodulin-dependent kinase II alpha isoform gene in skeletal muscle. Mol Cell Biol 16: 29-36.

25. Bertolino E, Wildt S, Richards G, Clerc RG (1996) Expression of a novel murine homeobox gene in the developing cerebellar external granular layer during its proliferation. Dev Dyn 205: 410-420.

26. Duncan FE, Moss SB, Schultz RM, Williams CJ (2005) PAR-3 defines a central subdomain of the cortical actin cap in mouse eggs. Dev Biol 280: 38-47.

27. Hong CS, Kwon SJ, Kim do H (2007) Multiple functions of junctin and junctate, two distinct isoforms of aspartyl beta-hydroxylase. Biochem Biophys Res Commun 362: 1-4.

28. Dinchuk JE, Henderson NL, Burn TC, Huber R, Ho SP, et al. (2000) Aspartyl beta -hydroxylase (Asph) and an evolutionarily conserved isoform of Asph missing the catalytic domain share exons with junctin. J Biol Chem 275: 39543-39554.

29. Kisielow J, Nairn AC, Karjalainen K (2001) TARPP, a novel protein that accompanies TCR gene rearrangement and thymocyte education. Eur J Immunol 31: 1141-1149.

30. Reiter JL, Threadgill DW, Eley GD, Strunk KE, Danielsen AJ, et al. (2001) Comparative genomic sequence analysis and isolation of human and mouse alternative EGFR transcripts encoding truncated receptor isoforms. Genomics 71: 1-20.
