# Supplementary material for: Comparative Analysis of mRNA Isoform Expression in Cardiac Hypertrophy and Development Reveals Multiple Post-Transcriptional Regulatory Modules
Source: PLoS One. 2011 Jul 22;6(7):e22391. doi: 10.1371/journal.pone.0022391 (PMC3142162; doi:10.1371/journal.pone.0022391)
Supplement: Table S1 — Datasets used in this study. (DOCX) [file pone.0022391.s009.docx]

**Table S1. Datasets used in this study.**

| **Dataset** | **Data Source*** | **Ref** | **Array Type** | **Strain** | **Description** |
| --- | --- | --- | --- | --- | --- |
| ED1 ED2 | GSE1479 | ** | Affymetrix Mouse Genome 430.2 | C57BL/6 | Embryonic development of mouse heart (E10.5, 11.5, 12.5, 13.5, 14.5, 16.5, 18.5), 3 samples per group. |
| PD1 PD2 | GSE11137 | [[3](#_ENREF_3)] | Affymetrix Rat Genome 230.2 | Sprague-Dawley | Postnatal development of rat heart (P1, P20, and P49), 3 samples per group. |
| EA | From authors | [[2](#_ENREF_2)] | Agilent custom splicing array | FVB | Embryonic and postnatal development of mouse heart (E17 and Adult). |
| LVH1 | GSE5500 | [[4](#_ENREF_4)] | Affymetrix Mouse Genome 430.2 | C57BL6/J-FVB/N | Hypertrophy induced by TAC (Sham and 1W TAC), 4 samples for Sham and 6 samples for TAC1w. |
| LVH2 | From authors | [[5](#_ENREF_5)] | Affymetrix Mouse Genome 430.2 | C57BL/6 | Hypertrophy induced by TAC (Sham and 1W TAC), 2 samples per group |
| LVH3  LVH4 | GSE24242 | *** | Affymetrix Mouse Exon 1.0 ST | C57BL/6 | Hypertrophy induced by TAC (Sham, 1W TAC, and 4W TAC), 2 samples per group |
| LVH5 | GSE7781 | [[6](#_ENREF_6)] | Affymetrix Mouse Genome 430.2 | C57BL/6 | Hypertrophy induced by TAC (Sham and 12W TAC), 3 samples per group |

*Samples with GSE accession numbers are from the Gene Expression Omnibus database.

**<http://www.cardiogenomics.org>

***This study.
