# Supplementary material for: Comparative Analysis of mRNA Isoform Expression in Cardiac Hypertrophy and Development Reveals Multiple Post-Transcriptional Regulatory Modules
Source: PLoS One. 2011 Jul 22;6(7):e22391. doi: 10.1371/journal.pone.0022391 (PMC3142162; doi:10.1371/journal.pone.0022391)
Supplement: Table S2 — Primers used in this study. (DOCX) [file pone.0022391.s010.docx]

**Table S2. Primers used in this study.**

| **Gene** | **Primer ID** | **Sequence** |
| --- | --- | --- |
| *Itgb1* | F1 | 5’-CCTACTGGTCCCGACATCAT |
|  | R1 | 5’-CAGAGACCAGCTTTACGTCCA |
|  | R2 | 5’-TAGGATTTTCACCCGTGTCC |
| *Fn1* | F1 | 5’-ATACCGTTGTCCCAGCTGTC |
|  | F2 | 5’-CGGAGAGAGTGCCCCTACTA |
|  | R1 | 5’-AGCTCTGCAACGTCCTCTTC |
| *A2bp1* | F1 | 5’-TGGCCCCAGTTCACTTGTAT |
|  | R1 | 5’-TCTGCACCATAAAATCCATCC |
|  | R2 | 5’-TTGCCATACACTGGCTCTTG |
| *Rbm9* | F1 | 5’-CTGGCTTCCCTTACCCAACT |
|  | R1 | 5’-CCGTAAAATCCGTCCTGGTA |
|  | R2 | 5’-GCGCTAATGATTGGTTCCTG |
| *Atp2b1* | F1 | 5’-GTGGCCAGATCTTGTGGTTT |
|  | R1 | 5’-CATCAATAAGGGGGATGTGC |
| *Abcc9* | F1 | 5’-CACCGAAGGTGGTGAGAACT |
|  | R1 | 5’-AACGAGGCAAACACTCCATC |
| *Tia1* | cUTR-F | 5’-CTCAAGGATTCCCTGTTGGA |
|  | cUTR-R | 5’-TAAATGCTGACGCACGAGAC |
|  | aUTR-F | 5’-GTGGGTCATGGAGCCAGTAT |
|  | aUTR-R | 5’-GACCATGGCTTCTGGGATAA |
| *Cugbp2* | cUTR-F | 5’-CCTGAAAAGCCCACACATTT |
|  | cUTR-R | 5’-CTGGGTGTGTGTTTGGACTG |
|  | aUTR-F | 5’-GTCAGCCGCTGCTTAAATTC |
|  | aUTR-R | 5’-TACGAGCCTGCAGATCACAC |
| *Anapc2* | cUTR-F | 5’-GAGCCTCTCTCTGGAGCGTA |
|  | cUTR-R | 5’-TGCTGTAGTCAGAGCCTGGA |
|  | aUTR-F | 5’-CCTCTATAGCCACCCAACCA |
|  | aUTR-R | 5’-ACTCTGAGCTGCCAACCCTA |
| *Cdk13* | cUTR-F | 5’-CGAGGCAGAGGGTTACCATA |
|  | cUTR-R | 5’-ATGCAAGACCCTTTCCACAT |
|  | aUTR-F | 5’-AGCTTAAGGTCTGCGGATGA |
|  | aUTR-R | 5’-GCCAAAAGACAGGCAGAAAG |
| *Ube2z* | cUTR-F | 5’-CCTAGCACCTGAGCACCTTC |
|  | cUTR-R | 5’-CAGTGATTGTGGGGACACAG |
|  | aUTR-F | 5’-TGTGGTGGGTTCCCTATCAT |
|  | aUTR-R | 5’-CGCCCAATCTTGAAAGCTAC |
| *Cstf1* | F | 5’-CAAGGACGGCTGCATCAA |
|  | R | 5’-GCTACAGAGTCTTTTCCACTTGAGA |
| *Cstf2* | F | 5’-CCCCAGGGATCCAGACA |
|  | R | 5’-AAGCAGCCTTCTCATGATCC |
| *Cstf3* | F | 5’-GAGGCCATGTCAGGAGAC |
|  | R | 5’-CATAAATCAATGTGCAAAACC |
