# Supplementary material for: Comparative Analysis of mRNA Isoform Expression in Cardiac Hypertrophy and Development Reveals Multiple Post-Transcriptional Regulatory Modules
Source: PLoS One. 2011 Jul 22;6(7):e22391. doi: 10.1371/journal.pone.0022391 (PMC3142162; doi:10.1371/journal.pone.0022391)
Supplement: Table S3 — Genes significantly regulated in hypertrophy and/or development. (DOCX) [file pone.0022391.s011.docx]

**Table S3. Genes significantly regulated in hypertrophy and/or development.**

| **Downregulated in LVH and DEV** |
| --- |
| *Alas2, Angpt1, Cdkn1c, Cdo1, Fdft1, Hmgcs2, Mpped2, Myl7, Rgs2,* |
| **Downregulated in LVH and no significant change in DEV** |
| *1700049G17Rik, 1810012P15Rik, 1810049H13Rik, 2610528E23Rik, 2700097O09Rik, A530016L24Rik, Abca4, Abcc9, Acacb, Acads, Acot1, Acsm5, Acy3, Adamts7, Akap1, Aldh5a1, Amd1, Anp32a, Apbb1, Arntl, As3mt, Atad3a, Cacna1h, Cacnb2, Car4, Ccbl2, Cdc26, Chd6, Cited2, Clasp1, Clcn3, Cpeb3, Cpt1b, Cpt2, Cry2, Cycs, Cytl1, D9Ertd402e, Dixdc1, Dnahc12, Dus4l, Dusp18, Dusp23, Efemp1, Efnb3, Egflam, Egln1, Ehhadh, Epha4, Ephx2, Fah, Fam73b, Fastk, Fbp2, Fign, Fitm1, Fkbp4, Fmc1, G0s2, Gpr22, Gstm7, Hadh, Hadha, Hmga1, Hnmt, Hook1, Hopx, Hspb1, Hspd1, Hsph1, Ift81, Iigp1, Il15, Impa2, Irs1, Irx2, Kcnh2, Kcnj3, Kcnk3, Klhl21, Lamb3, Lgals4, Lgr6, Lipe, Lrrc39, Mcee, Mgst3, Mitf, Mlycd, Mrpl37, Msrb2, Myl4, Mylk3, Ndufb6, Neo1, Nfyc, Nudt11, Ociad2, Oplah, Osgepl1, Pank1, Pcp4l1, Pde4a, Pdhb, Pdss2, Penk, Pex11a, Plk1s1, Plxnb1, Ppil1, Ppm1k, Ppm1l, Prox1, Qsox2, Rdm1, Retsat, Ric8b, Rpl19, Rtn2, Scn4a, Slc25a22, Slc25a26, Slc25a42, Slc36a2, Slc40a1, Slc41a3, Sord, Spcs2, Srebf1, Ssb, Stard10, Stat5a, Stk39, Suclg2, Sult1a1, Tfpi, Tfrc, Tmc7, Tmem205, Tmem69, Tnnt1, Tns1, Tppp, Trmt5, Ttll1, Ucp3, Uqcrb, Wnk2, Zfyve21,* |
| **Downregulated in LVH and upregulated in DEV** |
| *1700020C11Rik, 1700040L02Rik, 4833442J19Rik, 4930534B04Rik, A230051G13Rik, A2bp1, Acaa2, Acadvl, Acat1, Acot2, Acsl1, Actr3b, Adhfe1, Adra1b, Agpat9, Agtr1a, Aldh6a1, Amy1, Ano10, Aqp1, Aqp7, Arhgap20, Art3, Asb11, Bcat2, Bckdha, Bckdhb, Bcl6, Calr3, Car14, Cmbl, Cmtm8, Cox8b, Crat, Csdc2, Cyp4b1, Dbt, Dci, Decr1, Dgat2, Dnajc28, Doc2g, Ech1, Echs1, Efcab2, Enpp2, Entpd5, Fahd1, Fitm2, Fmo2, Fndc5, Gcat, Gm1337, Gna12, Gpcpd1, Gsn, Gstk1, Gstm1, H2-Q7, Hrc, Hsd11b1, Hsdl2, Itgb6, Ivd, Kcnd2, Kcnip2, Kcnv2, Ldhb, Ldhd, Lrg1, Lrrc3b, Lrrc4b, Lrtm1, Macrod1, Maob, Mapt, Mccc2, Mgl2, Myh11, Nampt, Nckap5, Nr3c2, P2ry1, Paqr9, Pdk2, Pdk4, Pdp2, Pfkfb1, Phkg1, Pink1, Pla2g5, Plin5, Ppara, Ppip5k2, Pptc7, Pxmp2, Rab17, Rap1gap2, Rxrg, Scn4b, Selenbp1, Slc22a3, Slc25a20, Slc27a1, Slc2a4, Slc38a3, Slc4a3, Stom, Tcea3, Tcp11l2, Tesc, Thrsp, Timp4, Tmod4, Tnfaip2, Tnni3k, Tuba4a, Tuba8, Vldlr, Ybx2, Yipf7,* |
| **Downregulated in DEV and no significant change in LVH** |
| *1110031I02Rik, 1200009O22Rik, 2010011I20Rik, 2810026P18Rik, 2900073G15Rik, 3632451O06Rik, 4930452B06Rik, 4930579G24Rik, 5730469M10Rik, 5930416I19Rik, Abcb10, Acpl2, Acvr2b, Adamts8, Afap1l2, Aif1l, Alad, Aldh18a1, Alg11, Ammecr1, Ano1, Aplp1, Aqp4, Arhgap1, Armcx1, Arrb1, Asb4, Atad5, Atp1a3, Atp6v1g2, Atp8a1, BC024479, BC052040, Bach2, Bambi, Bcat1, Bcl11a, Blvrb, Bmp2k, Bmp4, Bmp5, Bmper, Bpgm, Brca1, Car2, Casc4, Cblb, Cbr1, Ccdc107, Ccdc112, Ccdc23, Ccdc88a, Ccrl1, Cd8a, Cdc7, Cdca3, Cdk2, Cdk4, Cdkn3, Celsr1, Cenpn, Cenpq, Cep63, Chd7, Chek1, Chka, Chtf8, Cirbp, Cit, Cnpy4, Cntd1, Col9a1, Colec12, Cox6b2, Cpox, Creb5, Creld2, Cul4b, Cxcl12, Cyr61, Cytsb, D2Ertd750e, D3Bwg0562e, Dact3, Dck, Dctd, Dctpp1, Ddit4, Dhfr, Dhrs7, Dlg4, Dmxl1, Dnajc9, Dock7, Dok4, Dot1l, Dpp4, Drg1, Dsc2, Dsn1, Dstn, Dtymk, Dusp4, Dyrk2, E2f5, Ednrb, Eif2s3x, Emb, Emilin2, Eml4, Enc1, Epb4.1l3, Epb4.9, Epor, Erbb3, Etaa1, Exosc4, Ezh2, Ezr, Fads2, Fam110b, Fam117a, Fam122b, Fam184a, Fam49a, Fam76b, Fam83d, Fancb, Fat4, Fbxl7, Fbxo32, Fermt3, Flrt2, Fmr1, Foxm1, Foxp4, Frmd4b, Fzd1, Gadd45a, Galk1, Gas5, Gcfc1, Gch1, Gclm, Gins1, Glcci1, Gmnn, Gnai1, Gpr126, Grb10, Gria3, Gucy1a3, Gucy1b3, Gulp1, Gyg, H2-D1, Has2, Hbb-b1, Hebp1, Hells, Heph, Hira, Hmbs, Hpgd, Hsd17b12, Hspa5, Id1, Id2, Idi1, Ift20, Igf2, Igf2bp2, Igf2r, Ildr2, Ilf3, Irx4, Isg20, Itgb3bp, Itm2c, Jag1, Kank1, Kcna4, Kdelc2, Kdm2b, Kif18b, Kif2c, Klf11, Klhl13, Krt10, Lin9, Lmnb1, Lrp12, Lrrc16a, Lrrc42, Lta4h, Ltbp1, Maged1, Maged2, Mageh1, Marcksl1, Mast4, Matr3, Mbnl3, Mcm8, Mcts2, Mdm1, Mecom, Meis2, Mid1, Mier3, Mkrn1, Mllt3, Mmd, Mns1, Morc4, Msh6, Myef2, N4bp2, Nasp, Ncam1, Ncapd2, Ngfrap1, Nipbl, Nipsnap1, Npnt, Npr3, Nt5dc2, Nup85, Osgep, Pafah1b3, Palld, Papss2, Pawr, Pbx3, Pcsk5, Pctp, Pdcd4, Pdgfc, Pdia3, Pdk3, Peg3, Pfkl, Pfkp, Plagl1, Plagl2, Plk1, Plvap, Poc5, Podxl, Podxl2, Pola1, Polr3g, Ppbp, Ppp1r3d, Prkar2b, Psip1, Ptpla, Ptpn13, Ptprd, Pttg1, Purg, Qpct, Rad18, Rangap1, Rbm3, Rbm39, Rbm5, Rcn1, Reln, Rfc2, Rfc3, Rfc4, Rfesd, Rhd, Rnd2, Rnf2, Rsbn1, Rtn1, Runx1t1, Sass6, Satb1, Sema3c, Sema3d, Sept10, Sf3b5, Sfpq, Sgol2, Skp2, Slc22a17, Slc25a37, Slc2a1, Slc2a3, Slc38a1, Slc38a2, Slc38a4, Slc4a1, Smad6, Smtnl2, Snca, Sox9, Spc24, Sqle, Ssx2ip, Strn, Synm, Synpo, Tacc3, Tal1, Tbx3, Tbx5, Tceal8, Thbd, Tia1, Tmeff1, Tmem136, Tmem163, Tmem2, Tmem9, Tmpo, Tnik, Tnni2, Tspan2, Tspan33, Ttk, Tubb6, Tube1, Txndc11, Tyro3, Ube2o, Ube2t, Ung, Uros, Use1, Vcam1, Vegfc, Vrk1, Wasf1, Wdhd1, Wdr6, Wdr67, Wnt5a, Xpr1, Zfp142, Zfp280c, Zfp367, Zfp386, Zfp462, Zfp521, Zfpm1,* |
| **Upregulated in DEV and no significant change in LVH** |
| *1110007C09Rik, 1190002H23Rik, 1300014I06Rik, 2010002N04Rik, 2010317E24Rik, 2210020M01Rik, 2310002L09Rik, 4632428N05Rik, 6330409N04Rik, 8430408G22Rik, A230050P20Rik, A930038C07Rik, Abca8a, Abca9, Abcb4, Abcc8, Abhd1, Ablim1, Ablim3, Acadl, Acadm, Acadsb, Acn9, Acot11, Acot13, Acox1, Acsl6, Acss1, Acss2, Adamtsl4, Adamtsl5, Adcy9, Adm, Adora1, Adprhl1, Adrb1, Agl, Agpat2, Agtpbp1, Aif1, Akap6, Akr1c14, Alas1, Aldh1b1, Aldh1l1, Aldh2, Ampd3, Angptl1, Ank, Ank1, Aoc3, Ap1b1, Aplnr, Ar, Arl6ip5, Asb5, Asph, Atp1a2, Atp5k, Auh, B2m, BC004004, Bat5, Bdh1, Bhlhe40, Bmi1, Brp44, Brp44l, Bves, C1ra, C1s, C3, Cabc1, Cadm4, Casp12, Casq2, Cav2, Ccdc69, Ccl2, Cd14, Cd1d1, Cd200, Cd36, Cd83, Cdc37l1, Cdh13, Cdkl2, Cds1, Cdv3, Cebpb, Cenpv, Cfd, Chpt1, Cidea, Cisd3, Cish, Ckm, Ckmt2, Clcn4-2, Clec14a, Clec1a, Clic4, Clic5, Cmya5, Colec11, Commd3, Coq10a, Coq10b, Coq9, Corin, Coro6, Cox6a2, Cp, Cpe, Cpt1a, Cpxm2, Cst3, Ctsd, Ctsh, Cxcl9, Cyb5, Cyb5b, Cyfip2, Cygb, Cyp27a1, Cyp2d22, Cyp2e1, Cyp39a1, D16H22S680E, D4Bwg0951e, Dbp, Dcn, Ddo, Ddrgk1, Ddt, Ddx60, Dgat1, Dip2c, Dmpk, Dnaja4, Dnajb9, Dpep1, Dpt, Dtx3l, E130203B14Rik, Ebf2, Ebf3, Eef1a2, Eef2k, Efhd1, Egln3, Ehbp1, Eif4e3, Eltd1, Emp2, Eno3, Epas1, Epdr1, Ephx1, Erap1, Esam, Etfa, Etfb, F13a1, Fabp4, Fads1, Fads3, Faim3, Fam107a, Fam129a, Fam134b, Fam40b, Fam65b, Fas, Fcgrt, Fem1a, Fgf1, Fgf12, Fgf13, Fgf16, Fhl2, Fhod3, Fig4, Fli1, Fmo1, Frmd5, Fuca2, Fxyd1, Fyco1, Gabarapl1, Galntl4, Gas6, Gbe1, Gbp2, Gbp3, Gck, Gda, Gfpt2, Gga2, Ghr, Gimap4, Gja1, Glul, Gnb3, Gngt2, Golga4, Got1, Gpam, Gpc1, Gpd1, Gpr146, Gpr155, Gpsm1, Gpt, Gramd4, Grb14, Gsta3, Gstm2, Gstm4, Gsto1, Gvin1, Gyk, Gypc, H2-Ab1, H2-Eb1, Hadhb, Herc3, Herc5, Herpud1, Hfe2, Hhatl, Hibadh, Hic1, Hist1h2bc, Hk2, Hrasls, Hspb6, Hspb8, Htra1, Htra3, Idh2, Ifi203, Ifi205, Ifi35, Ifi44, Ifih1, Igfbp6, Igsf1, Igtp, Il10rb, Il13ra1, Immp2l, Irf1, Irf2, Irf9, Irgm1, Ispd, Itga7, Itga8, Itgb1bp2, Ivns1abp, Jam2, Kbtbd10, Kcnb1, Kcnd3, Kcnj8, Kcnk2, Kcnq1, Kif5b, Kifc3, Kitl, Klf15, Klf4, Klf9, Klhdc1, Klhl24, Krt80, L2hgdh, Lama2, Larp1b, Lbh, Lbp, Ldb2, Limch1, Lims2, Lmcd1, Lmod2, Lnx1, Lpin1, Lpl, Lrrc17, Lrrc2, Ly6a, Ly6c1, Ly6e, Lynx1, Lyve1, Mafk, Mal, Mamdc2, Man1c1, Maoa, Mb, Mcf2l, Me1, Me3, Megf6, Meox2, Metrnl, Mfn2, Mfng, Mgll, Mid1ip1, Mrap, Mras, Mreg, Mrps23, Ms4a4d, Murc, Mut, Myct1, Myh14, Myh6, Mylk, Myom2, Myot, Myoz2, Mypn, Napepld, Ncdn, Nceh1, Ndrg1, Ndrg2, Ndufa5, Ndufaf1, Ndufb10, Ndufs2, Nfix, Nlrp10, Nol3, Nos3, Notch3, Nov, Npas2, Nphp3, Nqo1, Nr1d2, Nr1h3, Nr3c1, Nr4a1, Nr4a3, Nrap, Nrarp, Nrbp2, Nt5dc3, Nt5e, Nudt18, Nudt7, Oas2, Oasl1, Obfc2a, Obscn, Ogn, Omd, Optn, Osbpl8, Oxct1, P2rx5, P2ry14, P2ry2, Pacsin3, Pam, Parp12, Parp14, Parp3, Parp9, Pcca, Pcdh12, Pcdh19, Pcsk6, Pde1c, Pde4dip, Pde8b, Pdk1, Pdp1, Per1, Per2, Per3, Pfkfb2, Pfkm, Pgam2, Phyh, Phyhd1, Pi4k2a, Pik3ip1, Pilra, Pitpnc1, Pla2g16, Pla2g2d, Plbd1, Plekhb2, Plekhf1, Plin3, Pln, Pltp, Pnpla2, Podn, Polr1e, Polr2k, Popdc3, Ppap2b, Ppargc1a, Ppl, Ppp1r3a, Prelp, Prkar2a, Prodh, Prrx1, Psen2, Psmb8, Psmb9, Ptgds, Ptgfr, Pxmp4, Pycard, Pygm, Rab11fip5, Rab20, Rab40b, Rapgef4, Rapsn, Rarres2, Rasgrp3, Rasl10b, Rassf3, Rbm20, Rbp7, Rcan2, Ret, Rftn1, Rgma, Rgs5, Rhot2, Rilpl1, Rnf125, Rnf144b, Rnf207, Robo4, Rom1, Rora, Ryr2, S100a1, S100a16, Sbk1, Scn5a, Scn7a, Scp2, Sdpr, Sdr39u1, Sema7a, Senp2, Sepp1, Sepx1, Serping1, Sestd1, Sfxn5, Sgcg, Sgk1, Sh2d4a, Sh3kbp1, Slc16a2, Slc1a1, Slc25a11, Slc25a29, Slc25a33, Slc28a2, Slc35f5, Slc4a1ap, Slc6a8, Slco5a1, Slfn5, Smap2, Smoc1, Smoc2, Smpd1, Smpdl3a, Sncg, Socs2, Sod2, Sod3, Sorbs1, Sox17, Sox18, Sp100, Spint2, Spop, Spsb1, Sqrdl, Srgn, St8sia4, St8sia6, Stat1, Stau2, Steap4, Sucla2, Syngr1, Tac1, Tapbp, Tasp1, Tcap, Tcf4, Tgfbr2, Tm4sf1, Tmcc3, Tmem108, Tmem140, Tmem143, Tmem182, Tmem204, Tmem38a, Tmem53, Tmem71, Tmem86a, Tmtc1, Tnfaip8, Tob1, Tor3a, Tpd52l1, Tppp3, Trak1, Trdn, Trf, Trim30, Trim63, Trip10, Tst, Ttc39b, Ttc7, Twf2, Txnip, Ube2ql1, Ugp2, Uqcrq, Ushbp1, Usmg5, Usp20, Vtn, Wfdc1, Xdh, Zbtb16, Zfp106,* |
| **Upregulated in LVH and downregualted in DEV** |
| *Acta1, Actn1, Akr1b8, Anln, Armcx2, Asns, Atad2, B3galnt1, Basp1, Bex1, Casp3, Casq1, Ccna2, Ccnb1, Ccnb2, Cd24a, Cd44, Cdca8, Cdh11, Cdt1, Chsy1, Cks1b, Cks2, Col18a1, Col5a2, Cotl1, Cpxm1, Csrp1, Csrp2, Ctgf, Cthrc1, D0H4S114, Dact1, Ddah1, Dkk3, Dpysl3, Dse, Dynll1, Ect2, Ell2, Fabp5, Fam111a, Fam64a, Fkbp10, Fn1, Frem2, Fscn1, Fstl1, Fzd2, Gatm, Gcnt1, H19, H2afz, Hn1l, Igf1, Itm2a, Kif11, Kif20a, Kif23, Kif26b, Klf10, Klhl4, Lig1, Loxl2, Lxn, Mad2l1, Mcm2, Mcm3, Mcm4, Mcm5, Mcm6, Mcm7, Mki67, Mmp14, Mtap1b, Mthfd2, Myh10, Myl1, Myof, Ncapg2, Ncaph, Ndn, Nppa, Nusap1, Odz3, Olfml2b, Olfml3, Pbk, Pdlim3, Pdlim5, Pdpn, Pf4, Phldb2, Prc1, Prelid1, Psat1, Ptgr1, Ptn, Racgap1, Rad51ap1, Rassf5, Rbl1, Rbp1, Rcn3, Rgs10, Rhou, Robo1, Rrm1, S100a4, Sept11, Serpine2, Sertad4, Sfrp1, Slit2, Smarca1, Smc2, Smc4, Stmn1, Tagln, Tcf19, Tgfb1i1, Tgfb2, Tgfbi, Thbs1, Tipin, Tnc, Tnfrsf12a, Top2a, Topbp1, Tpm2, Trip13, Tubb2b, Tubb5, Ube2c, Uchl1, Uhrf1, Vcan, Wbp5, Zdhhc2, Zwilch,* |
| **Upregulated in LVH and no significant change in DEV** |
| *2610029G23Rik, 2810474O19Rik, 5330426P16Rik, 5830433M19Rik, 6330406I15Rik, 9030425E11Rik, Abi3bp, Adam10, Adam9, Adamts1, Adamts2, Adamtsl2, Aebp1, Aldh1a1, Aldh1a2, Alox5ap, Ankrd1, Anpep, Antxr1, Anxa2, Anxa3, Apoe, Arhgdib, Arl4c, Arl6ip1, Arpc1b, Arsb, Atf3, Atp8b1, Atp8b2, Axl, BC028528, Baz1a, Bgn, Bmp1, Btg2, C1qtnf2, C3ar1, Cap1, Capg, Capza1, Carhsp1, Casp8, Ccdc34, Ccdc68, Ccdc80, Ccnd1, Cd34, Cd9, Cdc20, Cdkn1a, Cdkn2c, Cercam, Cilp, Ckap4, Clic1, Clu, Cmtm3, Cnksr1, Cnn3, Col1a1, Col1a2, Col3a1, Col4a1, Col4a2, Col5a1, Comp, Coro1a, Creb3l2, Ctla2a, Ctsc, Ctsk, Ctsz, Cyb561, Cyb5r3, Cybb, Cyth3, D930014E17Rik, Dap, Ddx39, Des, Dnmt1, Dok1, Ecm1, Efemp2, Efhd2, Egr2, Elovl1, Emilin1, Emp3, Emr1, Enah, Enpp1, Entpd7, F2r, Fam101b, Fam129b, Fam176b, Fam198b, Fam46a, Fat1, Fbln2, Fbln5, Fbn1, Fcgr1, Fcrls, Fhl1, Filip1l, Fkbp14, Flnb, Flnc, Fmod, Fos, Frzb, Fxyd5, Fxyd6, Gimap1, Glis2, Gnai3, Gnb4, Gpc6, Gpm6b, Gpr124, Gpx3, Gpx7, Gpx8, Grn, Gusb, Haus8, Hbegf, Hexb, Hmgn3, Hmox1, Hn1, Iah1, Icam2, Ier5, Ifi204, Ifi30, Ifitm2, Ift122, Il2rg, Il33, Il4ra, Incenp, Inpp5d, Iqgap1, Itga5, Itgb5, Itgbl1, Jun, Kcnj15, Kdelr3, Lamb1-1, Lamc1, Laptm5, Lass6, Leprel2, Lgmn, Lhfp, Lhfpl2, Limd2, Litaf, Loxl1, Loxl3, Lpar1, Lrp1, Lrp8, Lrrk1, Lsp1, Ltbp2, Lyn, Mall, Man2a1, Marcks, Mcam, Meox1, Mex3b, Mib1, Mllt11, Mmp2, Mmp23, Mpp1, Mrc2, Ms4a6c, Ms4a6d, Mt2, Mtap1a, Mxra7, Mxra8, Mybpc2, Myh9, Myl6, Myo1f, Myo5a, Nckap1l, Nes, Nfkbiz, Nid1, Nid2, Nipsnap3b, Nkd2, Nnmt, Nox4, Nrp2, Nuak1, Nucb2, Nup160, Nupr1, Oaf, Ostf1, Pabpc1, Pcna, Pcolce, Pdgfrl, Pdlim1, Pdlim2, Pecam1, Phlda3, Pi16, Pik3ap1, Plat, Plod2, Plp2, Pls3, Plxdc2, Pmepa1, Pmp22, Postn, Ppic, Prcp, Prdx4, Prelid2, Prkab2, Prkcb, Prkcd, Prkcdbp, Prss23, Ptgis, Ptk2b, Ptma, Ptplad2, Pxdn, Pycr1, Qsox1, Rab31, Rab4b, Rac2, Rai14, Rasl11b, Rcan1, Rell1, Rhobtb1, Rhoc, Rnf149, Rnf19b, Rpl15, Rrp12, Rtn4, Runx1, S100a10, Scarf2, Scpep1, Sdc1, Sec16b, Sec61b, Serp1, Serpinf1, Sfrp2, Sh3bgrl, Sh3bgrl3, Shisa3, Shisa4, Sla, Slbp, Slc25a24, Slc39a6, Slc5a3, Snx7, Soat1, Sox7, Sparc, Spp1, Stab1, Star, Stbd1, Stim2, Stx3, Sulf1, Sulf2, Sumf1, Svep1, Synpo2l, Tagln2, Tfpi2, Tgfb3, Tgfbr1, Tgif1, Tgm2, Thbs3, Thbs4, Timp1, Timp2, Tlr2, Tm6sf1, Tmem119, Tmem176b, Tmem184c, Tmem45a, Tmod3, Tmsb4x, Tnfaip6, Tns3, Tor1aip2, Tpm3, Tpm4, Trim47, Trps1, Tspan14, Tspan17, Tspan4, Tspo, Ttll7, Tuba1a, Tubb2a, Uck2, Vgll3, Vim, Vkorc1, Wipf1, Wisp1, Wsb1, Ywhah, Zfp354c, Zyx,* |
| **Upregulated in LVH and DEV** |
| *1500015O10Rik, 2810405K02Rik, Abra, Ace, Adamts12, Ankrd23, Anxa1, Anxa4, Aspn, C1qa, C1qb, C1qc, Cav1, Cd53, Cd74, Cfh, Clec4a3, Col14a1, Col15a1, Col6a1, Col6a2, Col6a3, Col8a1, Csf1r, Ctss, Cxcl16, Cxcr4, Cyp1b1, Egr1, Eln, Emp1, Fap, Fcgr3, Fgl2, Fndc1, Gng11, Gng2, H2-Aa, Higd1b, Ier3, Ifit2, Ifitm3, Igfbp7, Irf7, Irf8, Itih5, Lcp1, Lgals3, Lgals3bp, Lgals9, Ltbp3, Lum, Masp1, Matn2, Mfap4, Mfap5, Mgp, Mrc1, Ms4a6b, Msn, Mvp, Nppb, Oasl2, Osmr, Pcdh17, Pla2g4a, Ptprc, Rnasel, Rtp4, S100a6, Samd9l, Sat1, Scn1b, Scx, Serpine1, Sparcl1, Thbs2, Thy1, Tyrobp, Usp18,* |

The genes listed in this table correspond to the ones shown in Figure 1C. LVH, hypertrophy of left ventricle (only 1W TAC data was used). DEV, development.
