# Supplementary material for: Comparative Analysis of mRNA Isoform Expression in Cardiac Hypertrophy and Development Reveals Multiple Post-Transcriptional Regulatory Modules
Source: PLoS One. 2011 Jul 22;6(7):e22391. doi: 10.1371/journal.pone.0022391 (PMC3142162; doi:10.1371/journal.pone.0022391)
Supplement: Table S4 — Forty-five developmentally regulated skipped exons. (DOCX) [file pone.0022391.s012.docx]

**Table S4. Forty-five developmentally regulated skipped exons.**

| **Gene Symbol** | **Exon** | **EA** | **1W** | **4W** |
| --- | --- | --- | --- | --- |
| Dysf | chr6:+:84062135:84062176 | -0.42 | 0.83 | 0.70 |
| Asph | chr4:-:9551507:9551548 | 3.53 | -0.80 | -0.43 |
| Mtap2 | chr1:+:66364735:66364827 | -0.79 | 0.75 | -0.09 |
| G3bp2 | chr5:-:93138537:93138635 | 0.75 | -0.75 | -0.30 |
| Fn1 | chr1:-:71536868:71537137 | -1.38 | 0.71 | 0.13 |
| Fn1 | chr1:-:71546971:71547243 | -3.79 | 0.63 | NE |
| Sorbs1 | chr19:-:40353554:40353613 | -0.69 | 0.55 | 0.76 |
| Vegfa | chr17:-:45485858:45485929 | 0.55 | 0.51 | 0.24 |
| Slmap | chr14:-:25249387:25249476 | -0.61 | 0.50 | 0.73 |
| Itgb1 | chr8:+:131613073:131613153 | 1.75 | -0.50 | -0.16 |
| Ncor2 | chr5:-:125308798:125309025 | 0.65 | -0.49 | -0.22 |
| Anxa7 | chr14:-:19256010:19256075 | -1.20 | 0.46 | 0.28 |
| Tsc2 | chr17:-:24333824:24333952 | 0.44 | -0.44 | -0.54 |
| Atp2b1 | chr10:+:98448966:98449119 | -0.33 | -0.43 | -0.23 |
| Bat2l | chr2:+:32051272:32051353 | -0.25 | 0.43 | -0.06 |
| Sorbs1 | chr19:-:40448990:40449079 | 1.17 | 0.39 | 0.08 |
| Enah | chr1:-:183748272:183748334 | 0.53 | 0.39 | 0.20 |
| Sorbs1 | chr19:-:40375104:40375271 | 2.30 | 0.37 | 0.76 |
| Fam188a | chr2:-:12318878:12318929 | -0.69 | 0.37 | 0.07 |
| Slmap | chr14:-:25262972:25263094 | -0.48 | 0.29 | -0.07 |
| Gpr116 | chr17:+:42904327:42904446 | -0.75 | 0.26 | 0.05 |
| Ablim1 | chr19:-:57104380:57104484 | -0.16 | 0.24 | 0.23 |
| Capzb | chr4:+:138560937:138561049 | 0.56 | 0.23 | 0.18 |
| Ppfibp1 | chr6:+:146953979:146954071 | 1.07 | -0.23 | 0.06 |
| Sec24c | chr14:+:19464283:19464411 | -0.31 | -0.22 | 0.03 |
| Clip1 | chr5:-:123903037:123903234 | -1.19 | -0.22 | 0.30 |
| Mbnl2 | chr14:+:119539820:119539914 | -0.67 | 0.22 | 0.44 |
| Phka1 | chrX:-:98759815:98759991 | -1.49 | 0.21 | -0.22 |
| Tacc2 | chr7:+:130454164:130454289 | 1.05 | 0.19 | 0.36 |
| Myom1 | chr17:+:70987060:70987353 | -2.13 | 0.18 | 0.67 |
| Mpp6 | chr6:+:50106463:50106504 | 0.21 | -0.15 | -0.02 |
| Hnrnph2 | chrX:+:129949268:129949312 | -0.01 | 0.15 | -0.09 |
| Smyd1 | chr6:-:71164922:71164960 | 0.34 | -0.14 | 0.12 |
| Pbx3 | chr2:-:33998758:33998870 | 0.13 | 0.13 | 0.17 |
| Mbnl1 | chr3:+:60702612:60702665 | -1.68 | -0.10 | -0.20 |
| Aplp2 | chr9:-:30917126:30917293 | 0.31 | 0.10 | 0.10 |
| Csde1 | chr3:+:103169502:103169594 | 0.08 | -0.09 | 0.20 |
| Lrrfip1 | chr1:+:92933653:92933724 | -0.05 | -0.09 | 0.65 |
| Ttn | chr2:-:76766616:76766753 | 0.23 | 0.07 | 0.13 |
| Ktn1 | chr14:+:46647806:46647877 | 1.38 | -0.07 | -0.01 |
| Tmem134 | chr19:+:4131031:4131075 | -0.38 | -0.05 | -0.10 |
| Ankrd10 | chr8:-:11623669:11623830 | -0.11 | -0.03 | 0.41 |
| Svil | chr18:+:5060513:5060608 | -0.02 | 0.02 | 0.30 |
| Pum2 | chr12:+:8759281:8759517 | -0.77 | -0.02 | -0.04 |
| Vldlr | chr19:+:27303337:27303459 | -0.03 | 0.00 | -0.06 |

EA, 1W, and 4W are splicing index for EA, 1W TAC, and 4W TAC, respectively. Positive splicing index values indicate more inclusion and negative values indicate more exclusion. “NE” indicates expression is not detectable. Data are sorted according to the absolute value of 1W TAC.
