# Supplementary material for: Comparative Analysis of mRNA Isoform Expression in Cardiac Hypertrophy and Development Reveals Multiple Post-Transcriptional Regulatory Modules
Source: PLoS One. 2011 Jul 22;6(7):e22391. doi: 10.1371/journal.pone.0022391 (PMC3142162; doi:10.1371/journal.pone.0022391)
Supplement: Table S5 — Top 50 most significantly regulated exon skipping events in hypertrophy. (DOCX) [file pone.0022391.s013.docx]

**Table S5. Top 50 most significantly regulated exon skipping events in hypertrophy.**

| **Exon** | **Gene Symbol, ID, and Name** | **EA** | **1W** | **4W** | **Protein domains affected** | **Refs** |
| --- | --- | --- | --- | --- | --- | --- |
| chr19:11392852:11392908 | Ms4a7, 109225, membrane-spanning 4-domains, subfamily A, member 7 | NA | 2.39 | 0.44 | IPR007237:CD20-like, | - |
| chr3:54473600:54473683 | Postn, 50706, periostin, osteoblast specific factor | 0.07 | 2.03 | 1.86 | IPR016666:TGFb-ind_bIGH3/osteoblast_fac2, | [[7](#_ENREF_7),[8](#_ENREF_8)] |
| chr6:97290896:97290953 | Frmd4b, 232288, FERM domain containing 4B | NA | 1.98 | 0.21 | IPR000299:FERM_domain, IPR000798:Ez/rad/moesin, IPR019747:FERM_CS, IPR019748:FERM_central, IPR019749:Band_41_domain, IPR019750:Band_41_fam, | - |
| chr10:107677505:107677535 | Ppp1r12a, 17931, protein phosphatase 1, regulatory (inhibitor) subunit 12A | 1.08 | -1.88 | -0.14 | IPR017401:Pase-1_reg_su_12A/B/C_euk, | [[9](#_ENREF_9)] |
| chr16:44708078:44708140 | Cd200r1, 57781, CD200 receptor 1 | NA | -1.84 | -0.79 | IPR013106:Ig_V-set, | - |
| chr7:131229531:131229653 | Acadsb, 66885, acyl-Coenzyme A dehydrogenase, short/branched chain | NA | 1.74 | NA | IPR006090:Acyl-CoA_Oxase/DH_1, IPR013107:Acyl-CoA_DH_2_C, | - |
| chr8:88733109:88733217 | Phkb, 102093, phosphorylase kinase beta | -1.94 | 1.73 | 0.91 | IPR008734:PHK_AB, | [[10](#_ENREF_10)] |
| chr3:36035120:36035245 | Atp11b, 76295, ATPase, class VI, type 11B | NA | 1.72 | NA | IPR006539:ATPase_P-typ_Plipid-transl, | - |
| chr11:29606410:29608770 | Rtn4, 68585, reticulon 4 | -2.04 | 1.71 | 0.77 | IPR003388:Reticulon, | [[11](#_ENREF_11),[12](#_ENREF_12)] |
| chr11:60227862:60227924 | Atpaf2, 246782, ATP synthase mitochondrial F1 complex assembly factor 2 | NA | -1.66 | -1.85 | IPR011419:ATP12_ATPase-F1F0-assembly, | - |
| chr4:126605716:126605991 | Zmym6,100177, zinc finger, MYM-type 6 | 0.27 | 1.66 | 2.00 | IPR011017:TRASH, | - |
| chr8:56194966:56195094 | Wdr17, 244484, WD repeat domain 17 | NA | 1.62 | 1.50 | IPR011046:WD40_repeat-like_dom, | - |
| chr19:38245150:38245224 | Pde6c, 110855, phosphodiesterase 6C, cGMP specific, cone, alpha prime | NA | 1.61 | NA | IPR002073:PDEase_catalytic_dom, | - |
| chr2:3381539:3382198 | Suv39h2, 64707, suppressor of variegation 3-9 homolog 2 (Drosophila) | NA | -1.59 | -0.89 | IPR000953:Chromodomain, IPR001214:SET_dom, IPR003606:Pre-SET_Zn-bd_sub, IPR007728:Pre-SET_dom, IPR011381:Histone_H3-K9_MeTrfase, IPR016197:Chromodomain-like, | - |
| chr11:69333212:69333299 | Dnahc2, 327954, dynein, axonemal, heavy chain 2 | NA | 1.57 | NA | IPR013594:Dynein_heavy_N-1, | - |
| chr3:54473137:54473214 | Postn, 50706, periostin, osteoblast specific factor | 0.92 | 1.54 | 1.31 | IPR016666:TGFb-ind_bIGH3/osteoblast_fac2, | [[7](#_ENREF_7),[8](#_ENREF_8)] |
| chr9:82630165:82630225 | Irak1bp1, 65099, interleukin-1 receptor-associated kinase 1 binding protein 1 | NA | -1.53 | -1.39 | IPR007497:DUF541, | - |
| chr6:113551987:113552025 | Fancd2, 211651, Fanconi anemia, complementation group D2 | NA | -1.47 | -0.80 | IPR016024:ARM-type_fold, | - |
| chr3:95252817:95252880 | Sema6c, 20360, sema domain, transmembrane domain (TM), and cytoplasmic domain, (semaphorin) 6C | 0.18 | -1.47 | -1.44 | IPR001627:Semaphorin/CD100_Ag, | - |
| chr16:4630461:4630858 | Nmral1, 67824, NmrA-like family domain containing 1 | NA | -1.46 | NA | IPR003148:RCK_N, IPR008030:NmrA, IPR016040:NAD(P)-bd_dom, | - |
| chr16:56575662:56575721 | Abi3bp, 320712, ABI gene family, member 3 (NESH) binding protein | 0.50 | 1.45 | 0.09 | IPR000694, | - |
| chrX:38510993:38511103 | Stag2, 20843, stromal antigen 2 | -0.06 | -1.45 | -1.24 | IPR016024:ARM-type_fold, | - |
| chr3:66043059:66043205 | Ccnl1, 56706, cyclin L1 | -0.51 | 1.44 | 0.46 | IPR006670:Cyclin, | [[13](#_ENREF_13)] |
| chr8:59465078:59465193 | Fbxo8, 50753, F-box protein 8 | NA | -1.44 | -0.75 | IPR000904:Sec7, | - |
| chr7:141352860:141352915 | Chid1, 68038, chitinase domain containing 1 | NA | 1.43 | 1.37 | IPR001223:Glyco_hydro18cat, IPR011583:Chitinase_II, IPR017853:Glyco_hydro_catalytic_core, | - |
| chr1:152339490:152339840 | Hmcn1, 545370, hemicentin 1 | NA | -1.42 | -0.85 | IPR000742:EGF_3, IPR001881:EGF_Ca-bd, IPR006209:EGF, IPR006210:EGF-like, IPR013091:EGF_Ca-bd_2, IPR018097:EGF_Ca-bd_CS, | [[14](#_ENREF_14)] |
| chr7:101539983:101540071 | Clpb, 20480, ClpB caseinolytic peptidase B homolog (E. coli) | NA | -1.41 | -0.69 | IPR002110:Ankyrin_rpt, | - |
| chr8:56536026:56536218 | Gpm6a, 234267, glycoprotein m6a | NA | 1.41 | NA | IPR001614:Myelin_PLP, | - |
| chr1:171984671:171984718 | Uap1, 107652, UDP-N-acetylglucosamine pyrophosphorylase 1 | -0.10 | -1.40 | -0.67 | IPR002618:UDPGP_trans, | [[15](#_ENREF_15)] |
| chr3:131519188:131519298 | Papss1, 23971, 3'-phosphoadenosine 5'-phosphosulfate synthase 1 | NA | -1.37 | -0.58 | IPR002891:APS_kinase_C, | - |
| chr17:28420345:28420389 | Mapk14, 26416, mitogen-activated protein kinase 14 | 1.55 | 1.37 | 0.42 | IPR000719:Prot_kinase_cat_dom, IPR001245:Ser-Thr/Tyr-Pkinase, IPR002290:Ser/Thr_prot_kinase_dom, IPR011009:Kinase-like_dom, IPR017441:Protein_kinase_ATP_BS, IPR017442:Se/Thr_prot_kinase-like_dom, | - |
| chr9:22174515:22175209 | 9530077C05Rik, 68283, RIKEN cDNA 9530077C05 gene | NA | -1.36 | -0.52 | IPR002185:Dopa_D4_rcpt, IPR005819:Histone_H5, | - |
| chr3:54468341:54468421 | Postn, 50706, periostin, osteoblast specific factor | -1.53 | 1.36 | 0.87 | IPR016666:TGFb-ind_bIGH3/osteoblast_fac2, | [[7](#_ENREF_7),[8](#_ENREF_8)] |
| chr1:89605984:89606040 | Atg16l1, 77040, autophagy-related 16-like 1 (yeast) | -1.89 | -1.35 | -1.00 | IPR011046:WD40_repeat-like_dom, | [[16](#_ENREF_16)] |
| chr13:101226076:101226188 | Smn1, 20595, survival motor neuron 1 | 1.04 | 1.35 | 0.31 | IPR010304:Survival_motor_neuron, | - |
| chr2:154551352:154551399 | Raly, 19383, hnRNP-associated with lethal yellow | 0.10 | -1.33 | -0.31 | IPR017347:hnRNP_C_Raly, | [[17](#_ENREF_17)] |
| chrX:9469352:9469489 | Otc, 18416, ornithine transcarbamylase | NA | 1.32 | NA | IPR002082:Asp_carbamoyltransf_euk, IPR002292:Orn_carbamltrans, IPR006130:Asp/Orn_carbamoylTrfase, IPR006131:Asp_carbamoyltransf_Asp/Orn-bd, | - |
| chr1:137242335:137242397 | Ipo9, 226432, importin 9 | NA | -1.32 | -0.91 | IPR001494:Importin-beta_N, IPR016024:ARM-type_fold, | - |
| chr16:7307309:7307361 | A2bp1, 268859, ataxin 2 binding protein 1 | 1.39 | -1.32 | -0.30 | IPR017325:RNA-bd_9/Ataxin-2-bd, | [[18](#_ENREF_18)] |
| chr16:56109098:56109141 | Senp7, 66315, SUMO1/sentrin specific peptidase 7 | 0.32 | 1.30 | 0.97 | IPR003653:Peptidase_C48, | - |
| chr5:108859928:108860009 | Gak, 231580, cyclin G associated kinase | NA | -1.30 | 0.02 | IPR000719:Prot_kinase_cat_dom, IPR001245:Ser-Thr/Tyr-Pkinase, IPR002290:Ser/Thr_prot_kinase_dom, IPR011009:Kinase-like_dom, IPR017442:Se/Thr_prot_kinase-like_dom, | - |
| chr1:63080762:63080845 | Ndufs1, 227197, NADH dehydrogenase (ubiquinone) Fe-S protein 1 | NA | -1.29 | -1.80 | IPR015405:NuoG_C, | - |
| chr16:92533013:92533204 | Runx1, 12394, runt related transcription factor 1 | -0.64 | 1.28 | 0.83 | IPR013524:AML1/Runt_N, IPR016554:TF_Runt-rel_RUNX, | [[19](#_ENREF_19)] |
| chr2:69463865:69463999 | Bbs5, 72569, Bardet-Biedl syndrome 5 (human) | NA | -1.28 | -0.59 | IPR006606:BBL5, IPR014003:DM16_repeat, | - |
| chr5:116199678:116199803 | Cit, 12704, Citron | NA | -1.28 | -0.69 | IPR000533:Tropomyosin, IPR017405:Citron_Rho-interacting_kinase, | [[20](#_ENREF_20)] |
| chr8:26181071:26181147 | Adam3, 11497, a disintegrin and metallopeptidase domain 3 (cyritestin) | NA | -1.27 | -0.81 | IPR002870:Peptidase_M12B_N, | - |
| chr17:31953954:31954081 | Brd4, 57261, bromodomain containing 4 | 0.04 | -1.25 | -1.71 | IPR001487:Bromodomain, | - |
| chr6:49038512:49038646 | Igf2bp3, 140488, insulin-like growth factor 2 mRNA binding protein 3 | -3.18 | 1.24 | NA | IPR004087:KH, IPR004088:KH_type_1, IPR018111:KH_type_1_subgr, | - |
| chr2:118811885:118812013 | Rad51, 19361, RAD51 homolog (S. cerevisiae) | NA | -1.23 | -1.23 | IPR003583:Hlx-hairpin-Hlx_DNA-bd_motif, IPR010994:RuvA_2-like, IPR011941:DNA_recomb/repair_Rad51, IPR016467:DNA_recomb/repair_RecA-like, | - |
| chr2:120990710:120998813 | Mtap1a, 17754, microtubule-associated protein 1 A | NA | -1.23 | -0.94 | IPR000694:-, | - |

The “Exon” column contains information about chromosome and exon start and end positions based on transcription direction in the mouse mm8 genome build. Gene Symbol, ID, and Name are obtained from NCBI Gene database. EA, 1W, and 4W are splicing index for EA, 1W TAC, and 4W TAC, respectively. Positive splicing index values indicate more inclusion and negative values indicate more exclusion. NA indicates data not available. The “Protein domains affected” column indicates the protein domains associated with the regulated exons. The mapping of probeset to protein domain was retrieved from AltAnalyze [[21](#_ENREF_21)]. The “Refs” column shows references reporting the splicing events. Data are sorted according to the absolute value of 1W TAC.
