# Supplementary material for: Comparative Analysis of mRNA Isoform Expression in Cardiac Hypertrophy and Development Reveals Multiple Post-Transcriptional Regulatory Modules
Source: PLoS One. 2011 Jul 22;6(7):e22391. doi: 10.1371/journal.pone.0022391 (PMC3142162; doi:10.1371/journal.pone.0022391)
Supplement: Table S6 — Top 5 regulated AFE events in hypertrophy. (DOCX) [file pone.0022391.s014.docx]

**Table S6. Top 5 regulated AFE events in hypertrophy.**

| **Gene Symbol, ID, and Name** | **EA** | **1W** | **4W** | **Refs** |
| --- | --- | --- | --- | --- |
| Rtn4, 68585, reticulon 4 | -2.76 | 2.21 | 0.63 | [[11](#_ENREF_11),[12](#_ENREF_12)] |
| Dusp13, 27389, dual specificity phosphatase 13 | 0.17 | -1.89 | -0.61 | [[22](#_ENREF_22)] |
| A2bp1, 268859, RNA binding protein, fox-1 homolog (C. elegans) 1 | -3.82 | 1.88 | 0.73 | [[23](#_ENREF_23)] |
| Camk2a, 12322, calcium/calmodulin-dependent protein kinase II alpha | 0.90 | 1.83 | 0.61 | [[24](#_ENREF_24)] |
| Tgif1, 21815, TGFB-induced factor homeobox 1 | 0.07 | -1.55 | -0.41 | [[25](#_ENREF_25)] |

Numbers are difference in log_2_(U/D) between TAC and Sham, where U/D is ratio of probe set intensities between upstream (U) and downstream (D) 5’ terminal exons. Data are sorted according to the absolute value of 1W TAC.
