# Supplementary material for: Comparative Analysis of mRNA Isoform Expression in Cardiac Hypertrophy and Development Reveals Multiple Post-Transcriptional Regulatory Modules
Source: PLoS One. 2011 Jul 22;6(7):e22391. doi: 10.1371/journal.pone.0022391 (PMC3142162; doi:10.1371/journal.pone.0022391)
Supplement: Table S7 — Top 5 regulated ALE events in hypertrophy. (DOCX) [file pone.0022391.s015.docx]

**Table S7. Top 5 regulated ALE events in hypertrophy.**

| **Gene Symbol, ID, and Name** | **EA** | **1W** | **4W** | **Refs** |
| --- | --- | --- | --- | --- |
| Pard3, 93742, par-3 (partitioning defective 3) homolog (C. elegans) | 0.68 | -1.53 | -0.35 | [[26](#_ENREF_26)] |
| Asph, 65973, aspartate-beta-hydroxylase | -3.89 | 1.50 | 1.11 | [[27](#_ENREF_27),[28](#_ENREF_28)] |
| Arpp21, 74100, cyclic AMP-regulated phosphoprotein, 21 | 1.28 | -1.48 | -1.10 | [[29](#_ENREF_29)] |
| Egfr, 13649, epidermal growth factor receptor | -0.61 | 1.48 | 0.96 | [[30](#_ENREF_30)] |
| Ints8, 72656, integrator complex subunit | -1.57 | -1.42 | -1.15 | - |

Numbers are difference in log_2_(D/U) between TAC and Sham, where D/U is ratio of probe set intensities between downstream (D) and upstream (U) 3’ terminal exons. Data are sorted according to the absolute value of 1W TAC.
