# Supplementary material for: Comparative Analysis of mRNA Isoform Expression in Cardiac Hypertrophy and Development Reveals Multiple Post-Transcriptional Regulatory Modules
Source: PLoS One. 2011 Jul 22;6(7):e22391. doi: 10.1371/journal.pone.0022391 (PMC3142162; doi:10.1371/journal.pone.0022391)
Supplement: Table S8 — Skipped exons with Fox binding sites in the downstream intronic region. (DOCX) [file pone.0022391.s016.docx]

**Table S8. Skipped exons with Fox binding sites in the downstream intronic region.**

| **Gene Symbol** | **Exon** | **EA** | **1W** | **4W** |
| --- | --- | --- | --- | --- |
| Gyg | chr3:-:20315159:20315209 | -0.27 | 1.68 | 0.34 |
| Uap1 | chr1:-:171984671:171984718 | -0.10 | -1.40 | -0.67 |
| Gfpt1 | chr6:+:87026419:87026466 | 0.75 | -1.19 | -0.44 |
| Fmnl2 | chr2:+:52950726:52950851 | -0.43 | -0.93 | -0.92 |
| Mecom | chr3:-:30147379:30147562 | -0.83 | 0.86 | 0.72 |
| Asph | chr4:-:9551507:9551548 | 3.53 | -0.80 | -0.43 |
| Csnk1d | chr11:-:120781038:120781100 | 0.45 | 0.78 | 0.94 |
| Sorbs1 | chr19:-:40393356:40393457 | 0.77 | 0.70 | 0.61 |
| Fn1 | chr1:-:71546971:71547243 | -3.79 | 0.63 | NE |
| Rpn2 | chr2:+:157014664:157014711 | 0.18 | -0.62 | -0.17 |
| Myo1b | chr1:-:51713195:51713281 | -2.17 | 0.62 | 0.37 |
| Kcnd3 | chr3:+:105795018:105795074 | 0.00 | -0.62 | -0.41 |
| Ndel1 | chr11:-:68642365:68642399 | 1.08 | -0.61 | -0.28 |
| Scrib | chr15:-:75875177:75875251 | -0.42 | -0.60 | -0.41 |
| Dab2 | chr15:+:6376511:6377164 | -0.32 | 0.59 | 0.12 |
| Kitl | chr10:+:99510545:99510628 | -0.71 | 0.59 | -0.03 |
| Akap9 | chr5:+:3960403:3960456 | -0.77 | 0.58 | 0.03 |
| Srrm1 | chr4:-:134618392:134618521 | -0.35 | -0.57 | -1.06 |
| Sorbs1 | chr19:-:40414740:40414814 | 1.34 | -0.55 | 0.08 |
| Akap11 | chr14:-:77237094:77237177 | -0.34 | 0.55 | 0.22 |
| Fmnl3 | chr15:-:99146952:99147051 | 1.01 | 0.52 | 0.29 |
| Col4a3bp | chr13:+:97717693:97717770 | 0.04 | -0.52 | 0.19 |
| Dock7 | chr4:-:98485665:98485754 | -0.42 | -0.52 | -0.69 |
| Fnbp1 | chr2:-:30874994:30875146 | 0.37 | -0.52 | -0.34 |
| Rtn3 | chr19:-:7523344:7524504 | 1.56 | -0.51 | 0.40 |
| Golim4 | chr3:-:75988328:75988411 | 0.05 | -0.51 | -0.40 |
| A2bp1 | chr16:+:7207506:7207598 | -1.13 | -0.51 | -0.25 |
| Slmap | chr14:-:25249387:25249476 | -0.61 | 0.50 | 0.73 |
| Itgb1 | chr8:+:131613073:131613153 | 1.75 | -0.50 | -0.16 |
| Ppp1r12a | chr10:+:107656867:107657034 | -0.09 | 0.49 | 0.36 |
| Bcas3 | chr11:+:85360123:85360167 | -0.46 | 0.48 | 0.33 |
| Rnf130 | chr11:+:49942669:49942762 | 0.44 | 0.47 | 0.94 |
| Cacna1s | chr1:+:137920981:137921037 | 0.75 | -0.47 | -0.18 |
| Esco1 | chr18:-:10577636:10577757 | 0.00 | -0.47 | -0.36 |
| Myo9b | chr8:+:74287605:74287652 | 0.88 | 0.47 | 0.03 |
| Tsc2 | chr17:-:24333824:24333952 | 0.44 | -0.44 | -0.54 |
| Atp2b1 | chr10:+:98448966:98449119 | -0.33 | -0.43 | -0.23 |
| Tjp1 | chr7:-:65193043:65193282 | 1.46 | -0.42 | -0.08 |
| Fxr1 | chr3:+:34259710:34259801 | 0.48 | 0.40 | 1.65 |
| Sorbs1 | chr19:-:40448990:40449079 | 1.17 | 0.39 | 0.08 |
| AB030242 | chr5:-:23476199:23476496 | 0.18 | -0.39 | 0.12 |
| Ppfibp1 | chr6:+:146974031:146974063 | -0.40 | -0.35 | -0.39 |
| Lrp12 | chr15:-:39734613:39734669 | -1.63 | -0.35 | -0.74 |
| Epb4.1 | chr4:-:131235000:131235062 | 0.32 | -0.34 | -0.60 |
| Sipa1l2 | chr8:-:128309283:128309336 | 0.97 | -0.34 | -0.18 |
| Obscn | chr11:-:58831631:58831673 | 0.00 | -0.33 | -0.38 |
| Myl6 | chr10:-:127894669:127894713 | 0.64 | 0.31 | 0.46 |
| 0910001A06Rik | chr15:-:63787251:63787333 | -0.03 | 0.31 | 0.28 |
| Osbpl9 | chr4:-:108605813:108605881 | -1.20 | 0.30 | 0.20 |
| Pxdn | chr12:+:30565292:30565363 | -1.48 | 0.29 | -0.34 |
| Meis2 | chr2:-:115558251:115558345 | 0.25 | 0.28 | 0.38 |
| Smarca2 | chr19:+:26816949:26817044 | -1.37 | 0.28 | 0.48 |
| Ythdc1 | chr5:+:87895194:87895247 | 0.24 | 0.28 | 0.30 |
| Tsc22d2 | chr3:+:58516157:58516228 | 0.03 | -0.28 | 0.33 |
| Tra2a | chr6:-:49182389:49182694 | -0.32 | -0.27 | 0.21 |
| Sfrs6 | chr2:+:162624256:162624523 | -0.62 | 0.27 | -0.26 |
| Fam49b | chr15:-:63786266:63786348 | -0.40 | 0.27 | 0.20 |
| Adcyap1r1 | chr6:+:55420805:55420888 | -0.35 | -0.26 | -0.37 |
| App | chr16:-:84923690:84923746 | 0.86 | 0.26 | 0.18 |
| Reps1 | chr10:+:17797084:17797164 | -0.86 | 0.25 | -0.06 |
| Osbpl6 | chr2:+:76361025:76361099 | -1.09 | 0.24 | -0.04 |
| Sfrs3 | chr17:+:28767121:28767576 | 0.03 | -0.23 | -0.29 |
| Usp37 | chr1:-:74393122:74393187 | 0.98 | -0.23 | -1.84 |
| Pcnx | chr12:+:82847090:82847222 | 0.81 | -0.23 | 0.01 |
| Camk2d | chr3:+:126833507:126833595 | 0.66 | 0.20 | 0.80 |
| Atg16l1 | chr1:+:89606252:89606299 | 0.51 | -0.20 | -1.12 |
| Ablim3 | chr18:-:61944358:61944456 | -0.32 | 0.19 | 0.51 |
| Kif13a | chr13:-:46771635:46771739 | -0.86 | -0.18 | -0.64 |
| Trdn | chr10:+:32889579:32889638 | -1.36 | -0.18 | -0.48 |
| Rnf14 | chr18:+:38427630:38427781 | 0.15 | 0.18 | 0.08 |
| Git2 | chr5:-:114998347:114998436 | 0.23 | 0.17 | -0.13 |
| Fggy | chr4:+:95115499:95115587 | -0.20 | 0.16 | -0.44 |
| Wnk1 | chr6:-:119918304:119918588 | -0.83 | 0.16 | 0.20 |
| Tbc1d13 | chr2:+:29958682:29958722 | 0.00 | 0.16 | 0.00 |
| Sorbs1 | chr19:-:40429956:40430114 | -0.49 | -0.15 | -0.09 |
| Slc25a3 | chr10:-:90552006:90552130 | 0.03 | 0.14 | 0.43 |
| Ect2 | chr3:-:27339868:27339960 | 0.00 | 0.14 | 0.23 |
| Slc4a4 | chr5:+:90307325:90307421 | -0.12 | -0.14 | 0.37 |
| Gcap14 | chr14:-:35801535:35801667 | 0.46 | 0.13 | -0.20 |
| Limch1 | chr5:+:67272278:67272313 | 0.86 | 0.11 | 0.12 |
| Mprip | chr11:+:59588347:59588412 | -0.03 | 0.11 | 0.00 |
| Mbnl2 | chr14:+:119530894:119530947 | -1.43 | -0.09 | -0.25 |
| Ptprk | chr10:+:28251523:28251558 | 0.54 | 0.09 | -0.32 |
| Evi5l | chr8:+:4201934:4201966 | 0.50 | -0.09 | 0.06 |
| Lrrfip1 | chr1:+:92933653:92933724 | -0.05 | -0.09 | 0.65 |
| Dnm3 | chr1:-:164111165:164111216 | -0.42 | -0.07 | 0.09 |
| St7 | chr6:+:17855707:17855842 | -0.03 | 0.07 | 0.26 |
| AU040829 | chr11:-:64821649:64821700 | -0.40 | -0.06 | -0.21 |
| Lsm14b | chr2:+:179958366:179958482 | -0.02 | 0.06 | 0.05 |
| Epb4.1l1 | chr2:+:156216322:156216729 | -0.04 | 0.06 | 0.37 |
| Snap23 | chr2:+:120282263:120282355 | -0.36 | -0.06 | -0.48 |
| Mkrn1 | chr6:-:39330817:39331031 | -0.07 | -0.05 | 0.11 |
| Cacna1c | chr6:-:118596018:118596050 | 0.00 | 0.05 | -0.26 |
| Msra | chr14:-:63187939:63188043 | 0.36 | 0.04 | 0.07 |
| Rnf114 | chr2:+:167198252:167198402 | -0.32 | -0.04 | -0.23 |
| Ankrd10 | chr8:-:11623669:11623830 | -0.11 | -0.03 | 0.41 |
| Rps6kc1 | chr1:-:192534621:192534713 | -2.33 | -0.03 | -0.10 |
| Pola1 | chrX:-:89628310:89628403 | 0.75 | 0.03 | -0.41 |
| Clip1 | chr5:-:123899574:123899606 | 0.20 | -0.03 | 0.20 |
| Ptk2b | chr14:-:65117135:65117260 | 0.53 | -0.02 | -0.22 |
| Fgfr1op | chr17:+:8013693:8013752 | 0.00 | -0.02 | -0.08 |
| Arhgef12 | chr9:-:42791535:42791591 | 0.65 | -0.02 | 0.04 |
| Fchsd2 | chr7:+:101120389:101120460 | 0.76 | -0.01 | -0.05 |
| Efna5 | chr17:-:62298778:62298858 | 0.00 | 0.01 | 0.56 |

Numbers are splicing index values. “NE” indicates that expression is not detectable. Data are sorted according to the absolute value of 1W TAC.
